# Dynamical network analysis reveals long-range residue couplings at the pMHC interface underlying enhanced immunogenicity

**DOI:** 10.1038/s41540-026-00653-y

**Published:** 2026-01-28

**Authors:** Tom Resink, Benedetta Maria Sala, Renhua Sun, Xiao Han, Evren Alici, Flavio Salazar-Onfray, Tatyana Sandalova, Cheng Zhang, Hans-Gustaf Ljunggren, Adnane Achour

**Affiliations:** 1 Science for Life Laboratory, Department of Medicine, Karolinska Institute, Solna, Sweden; 2 Division of Infectious Diseases, Karolinska University Hospital, Solna, Sweden; 3 Centre for Hematology and Regenerative Medicine, Department of Medicine Huddinge, Karolinska Institute, Huddinge, Sweden; 4 Haematology Centre, Karolinska University Hospital, Huddinge, Sweden; 5 Disciplinary Program of Immunology, Institute of Biomedical Sciences, Faculty of Medicine, Universidad de Chile, Santiago, Chile; 6 Millennium Institute on Immunology and Immunotherapy, Faculty of Medicine, Universidad de Chile, Santiago, Chile; 7 Division of Systems Biology, Department of Protein Science, Science for Life Laboratory, KTH-Royal Institute of Technology, Stockholm, Sweden; 8 The Roger Williams Institute of Liver Studies, Faculty of Life Sciences & Medicine, School of Immunology & Microbial Sciences, James Black Center, King’s College London, London, UK; 9 Center for Infectious Medicine, Department of Medicine Huddinge, Karolinska Institute, Huddinge, Sweden

**Keywords:** Computational biology and bioinformatics, Immunology, Structural biology, Computer modelling

## Abstract

The interaction between a class I peptide-major histocompatibility complex (pMHC) and a T cell receptor (TCR) plays a central role in the elicitation of CD8^+^ T cell immune responses. As a result, considerable effort has been invested in understanding the structural, dynamic, and biophysical parameters that govern this recognition event, including designing altered peptide ligands (APLs) which seek to modulate the downstream signaling outcomes. However, dynamic links between modified peptide positions and distant residues have remained ill resolved until now. Using an integrative approach combining crystallographic ensemble and single models with atomistic molecular dynamics simulations and correlational analysis, we have established an approach that allows us to identify coupled dynamics between spatially distant residues at the pMHC interface. Furthermore, we constructed a network encoding the inter-residue couplings observed throughout the simulations. This computational workflow corroborates well with the functional and biophysical experimental data of our model system, and leads to novel insights regarding the differential immunogenicity of the closely related peptides analyzed in this study. Ultimately, we present an intuitive and comprehensive strategy for decoding the linked dynamics at the pMHC interface allowing for mechanistic insights into the biophysical bases governing immunogenicity.

## Introduction

The binding and recognition of a cognate class I major histocompatibility complex presenting a peptide antigen (pMHC) by a T cell receptor (TCR) is a critical step in the induction of CD8^+^ cytotoxic T lymphocyte (CTL) responses^1^. Class I pMHCs are exposed on the surface of all nucleated cells, each a trimer consisting of a heavy chain, β_2_ microglobulin (β_2_m), and a peptide bound within a peptide-binding cleft created by the α1 and α2 domains of the heavy chain. The surface formed by the solvent-exposed residues of the presented peptide and the α1 and α2 helixes creates the interface for TCR engagement^2^. Given the essential nature of the pMHC/TCR interaction in the elicitation of CD8^+^ CTL activity, considerable effort has been invested in understanding the mechanisms of antigen processing and presentation^3^, determining the structural and biophysical principles governing recognition^1,4^, and designing altered peptide ligands (APLs) to modulate the immune response^5^.

Structurally, the formation of a complementary surface between the TCR and pMHC is crucial for stable binding^6,7^. The presented peptide antigen^8,9^, which can be derived from oncogenic, autogenic, or pathogenic, including viral, sources, and the identity of the heavy chain allele^10–13^ contribute to the likelihood of TCR recognition^1,14^. Consequently, various pMHC properties have been used as predictors of immunogenicity, such as the thermal and kinetic stability of pMHCs^15–22^. Additionally, the sequence of the TCR complementarity-determining regions (CDRs) restricts the population of stimulatory pMHC molecules for a specific TCR^23,24^. TCRs are described as adopting a “canonical” diagonal docking topology when binding to pMHCs, and changes to the binding geometry can result in differential downstream responses^25,26^. Regarding biophysical interaction properties, it is challenging to devise a general rule that applies to all immunogenic pMHC/TCR pairings. Kinetically, the importance of specific association rates, dissociation rates, or the resulting dwell time differs between systems^27–32^. The thermodynamics of different stimulatory pairings can be driven by a combination of enthalpic and entropic contributions, as opposed to the historical model which dictated that generally unfavorable entropic effects were overcome by favorable enthalpic interactions^13,33–44^. Furthermore, other factors may also affect CD8^+^ CTL activation, such as the successful recruitment of CD8^45,46^ and CD3^47^ co-receptors or the presence of catch bonds at the TCR-pMHC interface, a requirement for the mechanosensing model of TCR recognition^48–52^. In practice, TCR-pMHC affinity generally correlates with the induction of CTL responses^27,44,45,53–62^. While exceptions to this rule exist, they typically violate other proposed requirements such as the canonical docking topology^26^ or the presence of catch bonds^63^.

In this study, we focused on a set of four highly related H-2D^b^-restricted peptides as a highly characterized model system to demonstrate the utility of our methods (Table 1). Within the context of murine lymphocytic choriomeningitis virus (LCMV) infection, a strong CTL response is induced against at least three immunodominant peptides^64–67^. 50% of this response will be targeted towards the nonameric epitope gp33 (KAVYNFATM) derived from residues 33-42 of the viral glycoprotein^68,69^. In this context, the sidechains of peptide residues p4Y and p6F are positioned centrally at the TCR-pMHC interface, engaging the CDR loops^70–73^. Moreover, residue p1K is also crucial for P14 TCR recognition specifically^72^. The selective pressure applied by CD8^+^ CTL activity promotes the emergence of common immune escape LCMV variants, which primarily manifest as single, relatively conserved, amino acid substitutions of p3V, p4Y, and p6F^67,74,75^. One such peptide variant, in which p4Y is mutated to phenylalanine (Y4F; KAV**F**NFATM), escapes recognition by altering the respective TCR contact^71,73^. The two other peptides investigated in this study are proline-APLs, where p3V has been substituted with a proline in gp33 (V3P; KA**P**YNFATM) and Y4F (PF; KA**PF**NFATM). Using circular dichroism and surface plasmon resonance measurements^62^, we have previously demonstrated that the p3P modification of gp33 and Y4F enhances the thermal stability of the pMHC, improves its 3D affinity for the P14 TCR, and promotes TCR internalization (Table 1). Importantly, this substitution of p3V to p3P does not significantly affect the overall pMHC conformation at the interface. Simultaneously, the ternary pMHC complex is stabilized through hydrophobic and CH-π interactions between p3P and Y159^62,76^, a residue which is nearly perfectly conserved among human and murine alleles^77,78^. Proline-APLs display enhanced immunogenicity compared to their wild-type counterparts in various systems^62,76,79–82^, and CD8^+^ CTL cross-reactivity is induced against wild-type peptides following APL vaccination^62,79,81^. Crystallographic structures of H-2D^b^-restricted gp33, Y4F, V3P, and PF highlight that specific H-2D^b^ (R62, E163, H155) and peptide (p1K, p6F) residues adopt a different conformation after p3P substitution. The altered conformation seemingly primes the pMHC for TCR engagement as the sidechain rotamers closely match those of the P14 TCR-bound structures. Despite the extensive functional, biophysical, and structural characterization of this set of peptides, a mechanistic insight into the conformational coupling of these residues remains elusive.

**Table 1:** pMHC stability and P14 affinity to MHC binding gp33 variants

Peptide	Tm (ºC)	K_D_ (μM)	TCR downregulation (%)
gp33 (KAVYNFATM)	53.7 ± 0.4	8.6 ± 0.4	69 ± 8
V3P (KAPYNFATM)	57.3 ± 0.4	5.6 ± 0.4	78 ± 4
Y4F (KAVFNFATM)	52.7 ± 0.4	ND	6 ± 1
PF (KAPFNFATM)	56.1 ± 1.1	80.3 ± 22	21 ± 4

The thermal stability of the pMHC was measured with circular dichroism (CD); T_m_ is the temperature where 50% of the molecules are denatured. The dissociation constant (K_D_) was determined from steady state surface plasmon resonance (SPR) data. TCR downregulation on P14 T cells exposed to 10^-8^ M peptide-pulsed RMA cells. Values in the table have been published previously^62^.

Given the marked reduction in the permitted and favored ranges of dihedral angles of proline compared to other amino acids, we set out to explore the ways in which the dynamics at the TCR-pMHC interface were altered following its introduction. Furthermore, other studies have begun to highlight the importance of localized residue flexibility, peptide dynamics, conformational rearrangements, and the overall pMHC free energy landscape in influencing TCR affinity and antigen presentation^2,83–94^. As such, we used an integrative approach, combining crystallographic ensemble refinement with molecular dynamics (MD) simulations and correlational analysis, to uncover dynamic relationships between the p3P substitution and other residues at the TCR-pMHC interface. We further expand on these analyses by encoding the observed correlational couplings as a network to intuitively and holistically explore the dynamics at the pMHC interface within the context of our chosen peptides. Overall, these network-based methods may facilitate the systematic assessment of dynamic mechanisms underlying the differential immunogenicity of closely related pMHCs.

## Results

### Similarities in packing between crystals allow for informative ensemble refinement

As the ensemble refinement is run using restraints derived from X-ray data^95^, it was important to analyze and evaluate the potential effects and artifacts introduced by the crystal geometry of each complex. The published structures^62,71,73^ of H-2D^b^/gp33, H-2D^b^/Y4F, and H-2D^b^/PF reveal similarities in crystal packing. Specifically, the three TCR-unbound crystals are in the same space group (Supplementary Table 1). An analysis of residues involved in polar interactions less than 4.0 Å apart (Supplementary Table 2) and visual inspection of the peptide-bound α1 and α2 domains further reveal the full extent of crystal contact conservation (Supplementary Fig. 1). All complex copies in each crystal form extensive contacts at the distal ends of the α1 and α2 helixes towards the C-terminal end of the peptide-binding groove. Two copies of each complex also form contacts through the N-terminal half of the α1 helix. Some crystal contacts are also found along the flexible loops connecting the antiparallel strands of the β-sheet that forms the base of the peptide cleft. The crystal packing around the peptides is also conserved between crystals, with two copies of each complex displaying more freedom around either p4Y/F or p6F (Supplementary Fig. 1). It should be noted that p4 in all unbound complexes was identified as a contact. However, we still expect to be able to gain some biological insight, beyond crystal artifacts, on the dynamics at this position as different p4Y/F rotamers are observed in each copy of each crystal. The arrangement of the peptide contacts does, however, hinder our ability to assess concerted conformational transitions observed in the peptide. We decided to consider all crystallographic copies of the peptide when assessing the conformational space occupied by each complex due to the differences in crystal contacts between copies.

In terms of the other complexes, H-2D^b^/V3P is not found in the same space group as the other unbound pMHC complexes (Supplementary Table 1), and only one copy is found in the asymmetric unit cell; thus, no alternative contact geometries were sampled. Fortunately, similar regions of the complex are involved in crystal contacts compared to the other TCR-unbound pMHC complexes (Supplementary Table 2**;** Supplementary Fig. 1). For completeness, H-2D^b^/V3P was included in the ensemble refinement analyses. The TCR-bound complexes H-2D^b^/gp33/P14, H-2D^b^/V3P/P14, and H-2D^b^/PF/P14 also revealed conservation of regions involved in crystal packing (Supplementary Tables 1, 3). As expected, no contacts involving the peptides were identified, and contacts at the interface were limited by the presence of the P14 TCR.

Given the similarities in crystal packing identified between the different published structures, we proceeded with the ensemble refinement of the previously published structures (Supplementary Table 1), which in most cases led to a slight improvement in the R-factors.

### The p3P substitution alters the conformational dynamics of MHC-restricted peptides

**Fig. 1:**
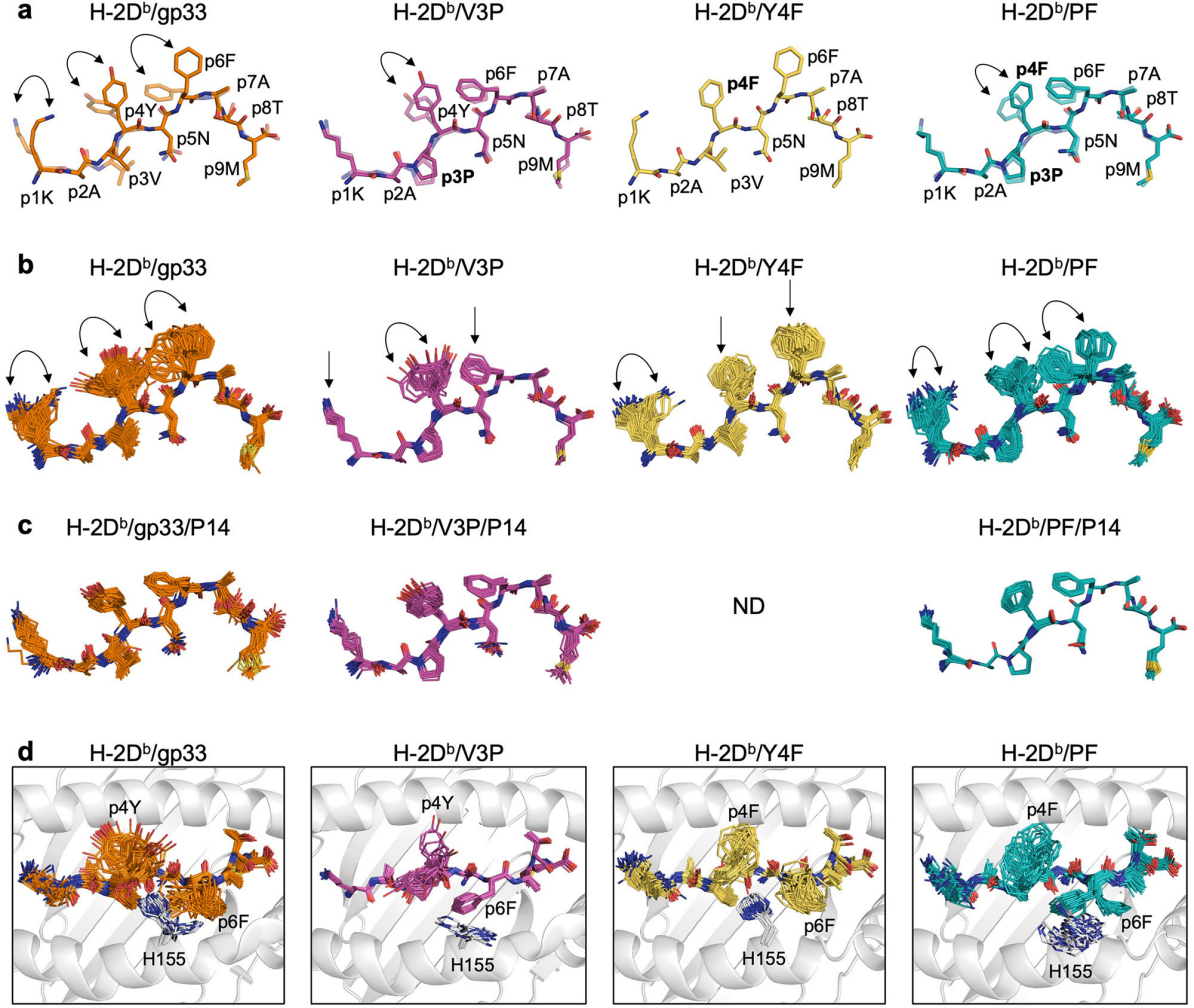
The conformational populations of gp33, V3P, Y4F, and PF peptides, calculated by ensemble refinement, unveil dynamic information from crystal structures. Stick representation of the peptides bound to the MHC. **a** Full-color peptides represent the peptide conformation before TCR binding in the crystallographic structure. In contrast, transparent peptides represent the conformation that the peptides assume after TCR binding. No crystallographic data are available for H-2D^b^/Y4F/P14. The arrows indicate the changes in sidechain conformation. **b** Polymorphic peptide conformations of gp33, V3P, Y4F, and PF of the TCR-unbound pMHC complex calculated by ensemble refinement. The arrows indicate variations in the conformation of the sidechain. **c** After TCR binding, the peptides rigidify, and all peptide ensembles assume the same restricted conformation. No crystallographic data are available for H-2D^b^/Y4F/P14. **d** Concerted and opposing motions between H155 and p6F sidechains were observed in the TCR-unbound pMHC complexes, particularly in H-2D^b^/gp33 and H-2D^b^/PF. H-2D^b^ (white) is shown in cartoon representation and the H155 sidechain is shown in stick representation.

The published crystal structures of TCR-unbound pMHC complexes H-2D^b^/gp33, H-2D^b^/V3P, H-2D^b^/Y4F, and H-2D^b^/PF have been previously compared with the corresponding TCR/pMHC complexes^62^. The peptide conformation in the TCR-unbound H-2D^b^/Y4F closely resembles that of the H-2D^b^/gp33 peptide. While, in H-2D^b^/V3P and H-2D^b^/PF, the p6F and p1K sidechains are observed in a different rotameric state, presumably due to the p3P substitution, leading to an overall conformation resembling the TCR-bound form (Fig. 1a). The binding of the P14 TCR results in conformational changes in the p4Y/F and p6F sidechains of gp33, V3P, and PF. Additionally, the sidechain of p1K in gp33 moves towards the N-terminal while it is already correctly positioned for P14 TCR engagement in V3P and PF (Fig. 1a).

Ensemble refinement allowed us to gain insights into the polymorphic characteristics of peptides (Fig. 1b, c). Residues p1K, p4Y/F, and p6F in the gp33 and PF peptides as well as residue p4Y in the V3P peptide display the greatest structural fluctuations. These sidechains explore different conformations, including those assumed after TCR binding in the crystal structure. In contrast, the p4F and p6F sidechains of the immune escape Y4F peptide ensemble do not sample a conformation similar to the TCR-bound conformation observed in other peptides, suggesting more restricted structural dynamics (Fig. 1b). Therefore, it appears that introducing p3P to the Y4F peptide in PF enables the peptide to sample a wider range of sidechain conformations, some of which are permissive to P14 TCR engagement, including the “prohibited” p6 flip. Our ensemble analyses imply that the lack of downward rotation of p4F in the Y4F peptide could be essential for hindering the formation of the TCR/pMHC complex. The TCR-bound pMHC ensembles display a decreased diversity of adopted peptide conformations, implying that TCR binding rigidifies the restricted peptide, stabilizing its conformation and reducing the accessible conformational space (Fig. 1c).

### Mechanistic insights into proline-altered dynamics are hindered by crystal contacts

In addition to comparing the presented peptides, we comprehensively analyzed the ensemble dynamics at the pMHC-TCR interfaces. The root-mean-square fluctuation (RMSF) profiles of each ensemble copy were compared (Supplementary Figs. 2, 3), in addition to various sidechain ensembles of MHC and TCR residues of interest (Fig. 1d**;** Supplementary Figs. 4-6). Most notably, a high degree of concerted motion was observed between the H155 and p6F sidechains, facilitated by their proximity in the 3D structure (Fig. 1d). While the sidechains of these two residues alternate between two main conformations in H-2D^b^/gp33, these same residues adopt only one conformation in H-2D^b^/Y4F. The observed inability of p6F to transition between sidechain rotamers, presumably due to steric hindrance induced by the H155 sidechain, may explain the lack of recognition by the P14 TCR. In contrast, the introduction of a proline in H-2D^b^/PF, recognized by P14 (Table 1), allows the formation of two main conformations for both p6F and H155 (Fig. 1d). This observation suggests a plausible mechanism by which the conformational sampling of the p6F sidechain is altered through the transmission of dynamics from p3P through the α2 helix and finally to p6F and H155. Interestingly, substituting p3V to a proline in gp33 is associated with only one conformation for p6F in H-2D^b^/V3P (Fig. 1d), leading to substantially increased recognition by the P14 TCR^62^.

Furthermore, the TCR-unbound complexes displayed a diverse distribution of RMSF values along the α1 and α2 domains (Supplementary Fig. 2). Increased disorder in loop regions was observed, particularly around residues 13-21 and 38-44. The introduction of p3P led to the rigidification of H-2D^b^, namely in the α1 and α2 helix residues surrounding p3P, but also in some loop regions. Similar overall rigidification of proline-APL pMHCs has been shown in other studies^76^. As expected^96,97^, the dynamics at the TCR-pMHC interface are considerably reduced after P14 TCR binding (Supplementary Fig. 3). While a relative reduction in dynamics in regions surrounding p3 is still observed, no relationship between dynamics at the TCR interface and affinity (Table 1) was identified (Supplementary Fig. 4), partly due to the lack of an available H-2D^b^/Y4F/P14 ternary structure. H155 assumes a stable conformation in all ternary complexes. Other residues, including E63, K66, E163, R75, R79, and K146, exhibit higher conformational variability in the H-2D^b^/gp33/P14 and H-2D^b^/V3P/P14 complexes compared to H-2D^b^/PF/P14 (Supplementary Figs. 5, 6). Unfortunately, the confirmation of the proposed transmission of dynamics and the overall reliability of the dynamic analysis were hindered by the prevalence of crystal contacts, primarily in the unbound pMHC ensembles, along the α1 and α2 helixes (Supplementary Tables 2, 3**;** Supplementary Fig. 1).

### MD simulations of the pMHC molecules agree with the crystallographic data

**Fig. 2:**
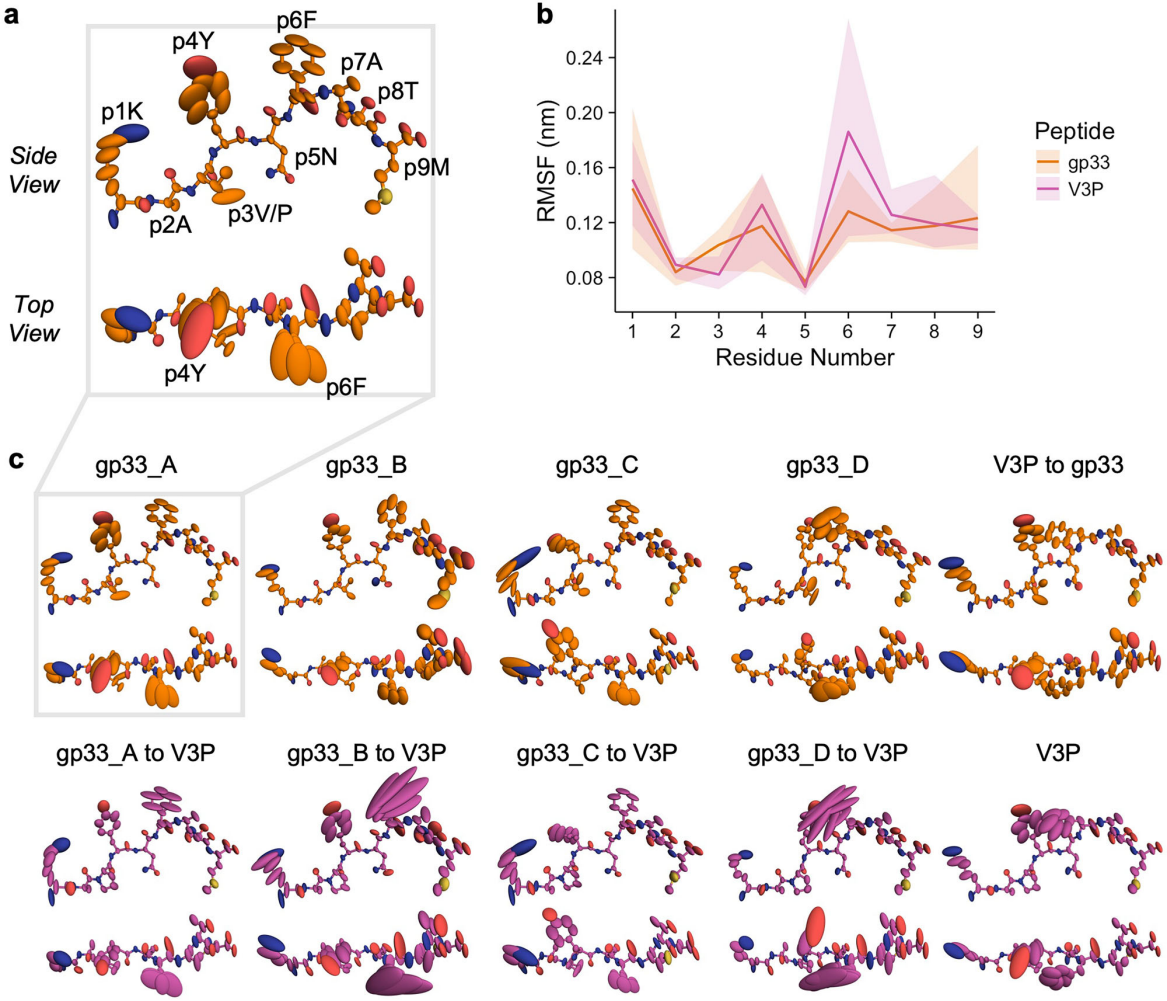
Overview of the conformational sampling of the gp33 and V3P peptides throughout the MD trajectories reveals that proline substitution leads to rigidification at p3 and increased dynamics at p6F. Stick and ellipsoid representation of MHC-bound gp33 (orange) and V3P (purple). Ellipsoids are scaled and shaped according to the anisotropic atomic temperature factors derived from each trajectory and mapped to their respective crystallographic models. **a** Residue labeling of the side and top views of the peptide as reference. **b** Average per-residue RMSF values over all trajectories for gp33 and V3P. The colored transparent envelopes depict the full range of observed trajectory RMSF values for each peptide. **c** Conformational sampling of the peptides observed in each trajectory. Each column represents a set of paired simulations. One derived from the initial crystallographic model and the other derived from an in silico mutation between p3V and p3P.

We ran unbiased MD simulations of each pMHC copy in the TCR-unbound crystallographic models to account for potential crystal artifacts in the ensembles. It must be noted that only the comparison between H-2D^b^/gp33 and H-2D^b^/V3P or H-2D^b^/Y4F and H-2D^b^/PF is stringently valid, as we only included an additional set of simulations where p3V was mutated to p3P or vice versa. Therefore, comparisons between H-2D^b^/gp33 and H-2D^b^/Y4F or H-2D^b^/V3P and H-2D^b^/PF do not adequately control for initial configuration biases. As a result, we cannot assess the dynamic effect of the p4Y to p4F mutation. We were, however, able to draw conclusions on the effects of p3P-altered ligands on antigen presentation and thus immunogenicity, given that the peptide conformations throughout each trajectory are comparable with the crystallographic ensembles and single models (Fig. 2**;** Supplementary Fig. 7). For clarity, a residue-labeling scheme has been provided for each set of trajectories (Fig. 2a**;** Supplementary Fig. 7a) We observed that the backbone dynamics and conformations are highly constrained and similar between all trajectories and that p1K, p4Y/F, and p6F sample the largest range of conformational space with their sidechains (Fig. 2c**;** Supplementary Fig. 7c). In the case of the gp33 and Y4F peptides, the p3V sidechain is also relatively more mobile than the rest of the peptide, with an increase in average per-residue RMSF value of 0.02 nm and 0.03 nm compared to their proline-altered counterparts, respectively. Critically, the per-residue RMSF data for p3V/P and p6F are the strongest correlates of pMHC immunogenicity (Supplementary Fig. 8).

Two main insights are gained from these trajectories regarding the observed biophysical and functional effects of the p3P substitution. Firstly, the overall dynamics, both backbone and sidechain, are remarkably comparable between p3V and p3P trajectories derived from the same crystallographic pMHC copy (Fig. 2c**;** Supplementary Fig. 7c). Most differences in average per-residue RMSF values were below 0.02 nm, and the largest difference of 0.05 nm was observed for p6F when comparing the H-2D^b^/gp33 and H-2D^b^/V3P trajectories. This may explain why overall pMHC melting temperature remains above 50 ^o^C, regardless of peptide (Table 1). Moreover, these similarities provide a basis for the observed P14 TCR cross-reactivity upon proline-APL vaccination via molecular mimicry^62^. Secondly, although p3P is consistently less dynamic than p3V, in some cases p4Y/F and p6F display greater RMSF values in the presence of p3P, which is observed both visually (Fig. 2c**;** Supplementary Fig. 7c) and in the per-residue RMSF data (Fig. 2b**;** Supplementary Fig. 7b). More specifically, p3V/P, p4Y/F, and p6F are the 3 residue positions displaying the largest average per-residue RMSF differences in H-2D^b^/gp33 and H-2D^b^/V3P. For H-2D^b^/Y4F and H-2D^b^/PF, these same residues in addition to p1K are among the 4 residues with the largest average RMSF differences. This was further analyzed statistically by considering each corresponding p3V and p3P trajectory as paired observations and performing an exact Wilcoxon signed-rank test on the per-residue RMSF data of p3V/P, p4Y/F, and p6F from all simulations. The uncorrected p-values of 0.013, 0.047, and 0.095 were obtained, with the alternative hypotheses that p3P is less dynamic than p4V, p4Y/F is more dynamic in p3P trajectories, and p6F is more dynamic in p3P trajectories, respectively. The link between p3V/P and p6F dynamics cannot be readily explained by altered conformational sampling of the peptide backbone (Supplementary Figs. 9-11). These observations, coupled with the previously published structures and presented ensemble refinements, may be sufficient to explain the differences in 3D TCR affinity and downstream P14 T cell activation^62^. Importantly, these MD trajectories, in which the influence of crystal contacts has been minimized, agree with the crystallographic ensemble and single model data.

As with the ensemble refinement, the H-2D^b^ dynamics were also assessed through visual inspection (Supplementary Figs. 12, 13) and RMSF profile comparison (Supplementary Fig. 14**)**. H155 displayed increased conformational sampling, particularly in trajectories where the p6F sidechain also sampled a greater range of conformations (Fig. 2c**;** Supplementary Fig. 7c). Visually, E163, which is positioned near p3, was consistently less dynamic in the presence of p3P. On the other hand, no dynamic relationship between R62 mobility and p3V to p3P mutation could be observed. It should be noted that the latter does not rule out the presence of an altered conformation upon introducing p3P^62^. The other residues of interest (E63, K66, R75, R79, K146) did not display consistent sampling differences when comparing the paired trajectories (Supplementary Fig. 13). This aligns with observations that the dynamics of these residues at the TCR-pMHC interface throughout the ensemble refinements did not relate to overall interaction affinity (Supplementary Figs. 4-6). One difference between the ensemble refinements and MD trajectories was identified between the overall per-residue RMSF profiles of H-2D^b^ (Supplementary Fig. 14). Specifically, the relative differences in H-2D^b^ dynamics in loop regions, such as the 38-44 and 86-93 loops, do not concur with the ensemble refinements (Supplementary Fig. 2). It cannot easily be ascertained whether this deviation is due to crystal artifacts, differential crystal packing, forcefield artifacts, stochastic assignment of initial velocities, or some other effect.

### Correlational analyses unveil dynamically linked clusters of residues

**Fig. 3:**
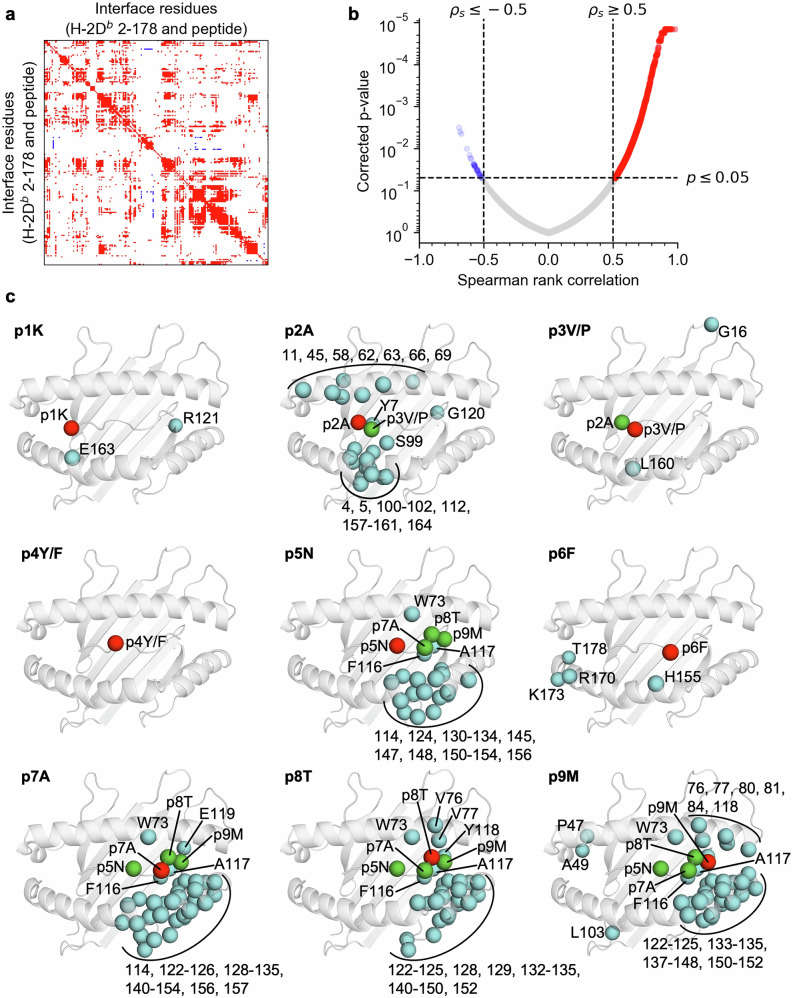
Mapping all significant pairwise correlates of peptide per-residue RMSF data onto the structure of H-2D^b^/gp33 yields spatially restricted clusters of dynamically similar residues. **a** Spearman rank correlation matrix depicting significant positive (red) and negative (blue) pairwise correlation between per-residue RMSF values at the interface. Nonsignificant values have been set to 0 (white). **b** Volcano plot of Spearman rank correlation and corrected p-values used to determine significant pairwise correlations. Note that the threshold of |ρ_s_| < 0.5 does not filter out any points in this dataset, when used in combination with a p < 0.05 threshold. **c** Significant pairwise correlates of each peptide residue (red) shown within their structural context. Correlates are colored depending on whether the residue is in the heavy chain (cyan) or the peptide (green). The backbone of H-2D^b^/gp33 (white) is shown in cartoon representation as reference.

Correlational substructure is visible in the pairwise Spearman rank correlation matrix of the per-residue RMSF data (Fig. 3a). A comparison of the significance threshold and Spearman correlation coefficient is also provided (Fig. 3b). Structural visualization is critical to identify groups of residues that cluster closely together in real space. Thus, the significant correlations of each peptide residue were mapped to the structure of H-2D^b^/gp33 (Fig. 3c). Consequently, the chance of deriving conclusions from false positives and spurious correlations is also reduced, as is likely the case between p1K and R121. While correlational analyses cannot typically infer directionality, our RMSF data is related over paired simulations, therefore, it seems likely that observed correlations will be partly due to the introduction of a proline in the region surrounding p3V/P.

It should be noted that no significant correlations were identified for p4Y/F (Fig. 3c). This may be explained by the solvent-exposed nature of the sidechain, resulting in considerably higher dynamics and freedom compared to surrounding residues, hindering strong correlations. Alternatively, it could be explained by the presently used simulation scheme, where p3P and p3V trajectories can be paired accordingly. We could investigate the dynamic effects of p4 mutations at the interface by also generating paired p4Y and p4F trajectories. Although it seems unlikely the results would be as striking compared to those of the p3P substitution given the solvent-exposed nature of the residue. In other words, we cannot adequately explain the lack of correlation with p4Y/F. As an addendum, if this lack of dynamic coupling is biophysically representative, it would suggest that the p3P subverts Y4F-dependent immune evasion by enhancing P14 TCR affinity through p6F, rather than altering the dynamics at p4Y/F.

On the other hand, the dynamics at the main anchor residues for H-2D^b^, p5N and p9M^79,98^ correlated strongly with numerous residues of H-2D^b^. Although coupled residues are also present in the α1 helix and at the base of the peptide cleft, the largest cluster is found in the α2 helix. This relationship can be extended to residues p7A and p8T, which, despite not being anchor residues, also show extensive correlation with the same MHC regions. In the case of H-2D^b^/gp33 and related peptides, p2A and p3V/P act as secondary anchor residues^44,71,73^. Our data corroborates this as p2A and p3V/P are correlated, and p2A is further linked with multiple residues along both α1 and α2 helixes (Fig. 3c). Notably, the dynamics of R62, directly, and E163, through proximity, are associated with those of p2A and p3V/P. These H-2D^b^ residues were identified to have altered conformations after the introduction of p3P and form important contacts with the P14 TCR^62^. H155, another critical residue for TCR contacts, correlates with p6F, which closely matches our observations from the ensemble refinement (Fig. 1d). While H155 and p6F do not strictly cluster with the directly surrounding peptide and heavy chain residues, changes to their backbone dynamics will bias the propensity for sampling specific sidechain conformations^99^. Additionally, p3P substitutions are stabilized by Y159^62,76^, and our data shows that the dynamics for residues 157-161, p2A, and p3V/P are related.

The coupling of residue dynamics can also be investigated using agglomerative hierarchical clustering on rank correlation distance of the per-residue RMSF data (Supplementary Fig. 16), instead of assessing significant pairwise Spearman rank correlations. This yields very similar results, in which p3V/P and p2A cluster with Y159 on the α2 helix, H155 and p6F cluster with each other, and the rest of the α2 helix clusters with p5N and p7A (Supplementary Fig. 17). These findings begin to paint a picture of how the p3P substitution can affect the conformational sampling of p6F, and ultimately whether the pMHC can be recognized by the P14 TCR. In particular, we propose that the altered dynamics at p3P are predominantly transmitted via the α2 helix to H155 and p6F. While promising, the methods presented thus far adopt a relatively reductionist approach to investigating pMHC dynamics at the interface. It would, therefore, prove valuable to encode the correlation of residue dynamics as a network to probe interface dynamics holistically using graph theory.

### Dynamic correlations can be used to generate a biophysically relevant network

**Fig. 4:**
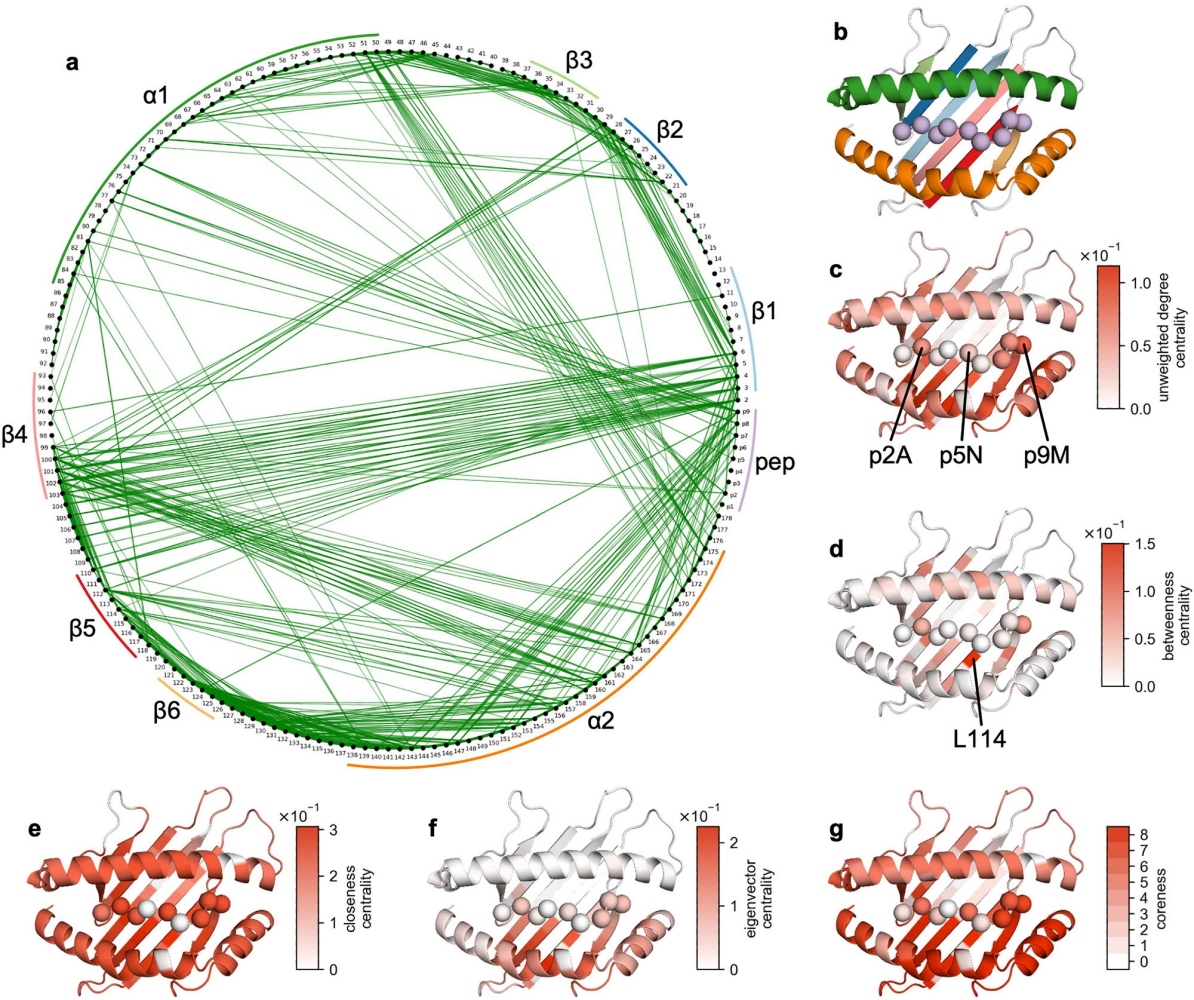
A biophysically relevant network can be derived from the correlation matrix revealing residues playing a central role in interface dynamics. **a** The network encoding interface dynamics, where each node (black) represents an interface residue and edges (green) are significant correlations between residues whose alpha-carbon distance does not surpass 10 Å. Edge transparency and width are scaled according to |ρ_s_|. Secondary structure features of the interface have been labelled. **b** Secondary structure features of the H‑2D^b^/peptide interface colored according to the same label colors as in **a**. Various informative network centrality measures can be mapped to structure: **c** unweighted degree centrality, **d** betweenness centrality, **e** closeness centrality, **f** eigenvector centrality, and **g** coreness. Plots showing centrality measures per residue are provided (Supplementary Fig 19).

Networks were constructed by defining each residue as a node in the network and deriving the edge weights from the matrix of significant Spearman rank correlations (Fig. 3a). We devised three path-based validation metrics to validate whether networks derived from the correlation data yielded biophysically relevant observations: F_1_, F_2_, and F_3_ (**Methods**). F_1_ accounts for uncharacteristically distant couplings observed in all shortest paths. This is achieved by calculating the proportion of shortest paths in the network where any edge travels a larger through-space distance than the initial distance. F_2_ and F_3_ provide related measures of the proportion of shortest paths that converge directly to their respective target nodes. They measure the proportion of shortest paths where the through-space distance increases at any node on the path relative to all previous nodes or the initial node respectively. F_3_ was designed to partially address the issue of edges, which diverge from the target node yet display high edge betweenness, being counted multiple times when calculating F_2_. These metrics and the proportion of nodes in the largest connected component were calculated for each network derived from a 2D scan of network construction parameters (Supplementary Fig. 18). Based on a compromise between minimizing F_1_ and maximizing the connected residues in the network, we constructed the network by only considering significantly correlated residues whose inter-residue C_α_ distance was less than 10 Å (Fig. 4). We decided not to incorporate a distance-based cost into the edge weight metric to reduce biases introduced in network construction. Furthermore, the validation metrics of networks with a distance threshold of 10 Å only improved marginally when including distance in the edge weight (Supplementary Fig. 18).

An analysis of the topological properties of our biophysically relevant network produced expected observations and novel insights regarding this set of H-2D^b^-restricted gp33-associated peptides (Fig. 4**;** Supplementary Fig. 19). Importantly, edges are observed between residues in neighboring secondary structure features as expected (Fig. 4a, b). Moreover, not every neighboring residue is linked, highlighting the discriminatory capacity of the network in identifying dynamically coupled residues through correlational metrics rather than reproducing a simple pairwise distance matrix. When considering the unweighted degree centrality (Fig. 4c) of each node, p2A, p5N, and p9M are amongst the most connected peptide residues paralleling their role as anchor positions^44,71,73,79,98^. Residue L114 displays the highest betweenness centrality (Fig. 4d), indicating it sits on the largest proportion of shortest paths in the network. It may, therefore, play a likely central role in transmitting dynamics throughout the interface. In class I pMHCs, residue 114 forms part of the F pocket for peptide binding and is found in a region whose dynamics have been linked to complex stability and peptide loading efficiency^70,73,90,100–102^. In terms of the closeness centrality of each residue (Fig. 4e), most of the complex is similarly central, aside from loop regions and nodes outside of the largest connected component, as would be expected for a globular protein. Both the eigenvector centrality (Fig. 4f), which favors high degree connectivities with other highly connected nodes, and k-coreness (Fig. 4g) of each residue identify the α2 helix as forming an influential and core part of the network. It is not immediately evident whether the latter observation is due to conserved dynamic patterns at the pMHC interface, a system-specific result of analyzing paired p3V and p3P trajectories, or a combination thereof. Nevertheless, these findings reflect the ability of our constructed network to capture the biophysically relevant coupled dynamics at the pMHC interface. For completeness, a network derived directly from the correlation matrix, without any modification or transformation, and its corresponding topological analysis is also available (Supplementary Figs. 20, 21).

### Network analysis unveils long-range coupling between residues p3 and p6

**Fig. 5:**
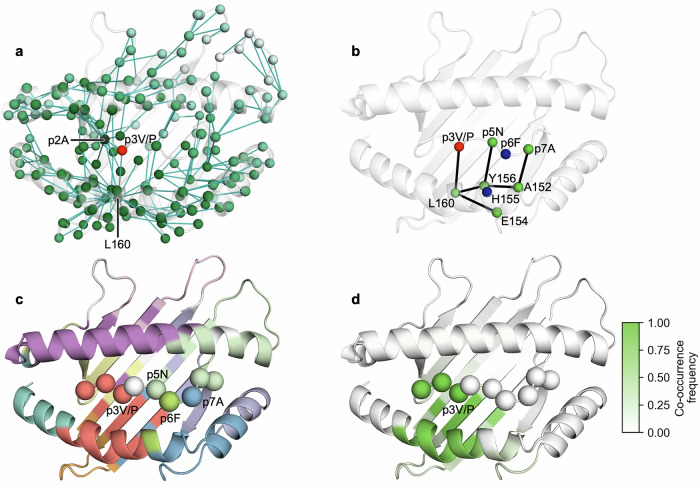
Shortest path analysis and Louvain community detection indicate that the dynamics of p3V/P are coupled through the α2 helix with the residues surrounding p6F and H155. **a** All shortest paths from p3V/P (red) to every other node in the largest connected component of the network. The dynamic signal of p3V to p3P mutation is firstly transmitted to p2A and L160 before propagating to the rest of the interface, primarily through the α2 helix. Residues are colored according to their shortest path distance from p3V/P, from green to white. Edges (teal) traversed by the shortest paths are shown. **b** Shortest paths from p3V/P to the residues neighboring p6F and H155. **c** Most frequent Louvain community detection solution obtained, representing 7.85% of solutions from 1 x 10^6^ iterations. Each community of residues has been depicted in a different color; residues that are not in any community are shown in white. The first three peptide residues cluster with the part of the α2 helix which neighbors communities containing p6F and p7A. **d** Community co-occurrence frequency of each residue with p3V/P over all Louvain community detection iterations. This demonstrates that p3V/P consistently clusters with residues on the α2 helix.

Using a network to describe pMHC dynamics opens the door to a suite of potential network analyses to probe the structural and biophysical properties of the pMHC interface. We present some examples such as shortest path analysis and Louvain community detection (Fig. 5). By mapping all shortest paths originating from p3 onto the structure of H-2D^b^/gp33, we observe that the dynamics at p3V/P are correlated to those of p2A and L160, the latter on the α2 helix, before being transmitted across the whole interface. This transmission occurs primarily through the α1 and α2 helixes (Fig. 5a). A connected subgraph of this network (Fig. 5b) shows that the shortest path of dynamic coupling from p3 to p6 is through the α2 helix. Namely, p3P transfers its dynamics to L160, likely via its interaction with Y159^62,76^, before this propagates to the residues surrounding p6F and H155. It should be noted that p6F and H155 are not part of the largest connected component of the biophysically relevant network. However, altered backbone angle distributions could explain the increased conformational sampling of the p6F sidechain^99^. In fact, the backbone and Ψ angles of p6F and the Φ angle of p7A display some of the largest deviations in conformational distribution after the introduction of p3P (Supplementary Figs. 9f, 10f, 11f). Furthermore, community detection through the Louvain algorithm corroborates the dynamic coupling between p3V/P and the α2 helix, as seen in the most frequent solution obtained in 7.85% of iterations (Fig. 5c), and community co-occurrence frequency of each residue with p3P (Fig. 5d). The latter was calculated as we postulated that the low recurrence of the most frequent solution was due to several residues on community fringes that would cluster inconsistently rather than fully or mostly unique community structures with each iteration. This was confirmed by the high community co-occurrence frequency of p1K, p2A, p3V/P, and the region of the α2 helix containing Y159 and L160.

## Discussion

The immunostimulatory potential of class I pMHC molecules is governed by a combination of the complex’s composition, structure, stability, and dynamics^1^. Critically, the surface properties and dynamics at the TCR interface are altered by the loading of different peptides in the pMHC peptide-binding cleft. These dynamics extend beyond local fluctuations, resulting in multiple conformational states within the complexes^42,89,97,103^.

In the context of both the unbound pMHC and P14 TCR-bound pMHC, the H-2D^b^-restricted gp33, V3P, Y4F, and PF peptides have been extensively structurally, biophysically, and functionally characterized by our group^22,44,62,71–73,104,105^. The free energy surface and conformational space inhabited by the peptides in H-2D^b^/gp33, H-2D^b^/V3P, H-2D^b^/gp33/P14, and H-2D^b^/V3P/P14 were also investigated using MD simulations^106^. Our present results indicate that altered dynamics at the third peptide residue upon p3P substitution can result in long-range modulation of conformational sampling of p6F through dynamic coupling via the α2 helix. While this mechanistic insight has been missing for this model system of peptides, the dynamic interplay between the peptide and surrounding α1 and α2 domains is well established^2,83–92^. Despite our analyses being primarily centered on the unbound pMHC, they corroborate well with the existing structural and functional models, which indicate that p3P-APLs prime the complex for engagement by the TCR^62^. In particular, we propose that the increased sampling of TCR-permissive sidechain conformations of p6F, observed in the crystal structure, ensemble refinement, and MD trajectories, is facilitated through long-range dynamic coupling with p3P. This may explain why V3P and PF act as a superagonist or a partial agonist respectively, in terms of P14 T cell and endogenous CD8 T cell responses compared to the full agonist gp33 or the null immune escape peptide Y4F^62^.

As mentioned, no correlational linkages were observed between p4Y/F and any other residues. However, it is not clear whether this is due to the MD simulation pairing scheme or the increased dynamics of the solvent-exposed p4Y/F sidechain. Furthermore, the mechanism driving Y4F-dependent immune escape seems to be caused by the altered electrostatic surface potential at the interface and loss of critical P14 TCR contacts^62,71,73^, rather than the dynamic modulation of neighboring residues. The Y4F modification abolishes the possibility of establishing hydrogen bond interactions as well as hydrophobic interactions with the sidechain of the P14 residue R97β and additional hydrogen bond interactions with the H-2D^b^ residue E163 and the TCR residue Y101α^62^. As such, it seems unlikely that this observation detracts from our findings. Additionally, it remains to be seen whether these long-range dynamic couplings of interfacial residues are conserved among other proline-APL systems^76,79,82^. Other MD and crystallographic studies of the H-2D^b^-restricted melanoma-associated proline-APL of gp100 and Trh4 demonstrated that the p3P substitution results in overall rigidification at the interface^76,82^.

We have demonstrated that the present MD protocol aligns well with single model and ensemble crystallographic observations of peptide dynamics and specific H-2D^b^ residues that are key for TCR recognition. However, dynamic links were largely obscured by the presence of crystal contacts along the α1 and α2 helixes. Previous ensemble refinement studies of pMHC molecules circumvent the issue by only analyzing human class I pMHC ensembles with resolutions better than 3 Å that displayed the largest RMSFs at the interface^84^. This approach cannot completely account for contacts and would reduce our agency in choosing which system to investigate. Serendipitously, the space groups and arrangements of crystal contacts of the H-2D^b^-restricted gp33, V3P, Y4F, and PF crystals allowed for the validation of MD trajectories to establish a reasonable simulation protocol. It may now prove interesting to adapt the combined MD and dynamical network analysis workflow to other proline-APLs, such as those derived from the H-2D^b^-restricted cancer-associated gp100^79^ or Trh4^80,82^ peptides. These were previously inaccessible for extensive ensemble refinement analyses due to their unfavorable crystallographic contact parameters.

Using the per-residue profiles of interface dynamics, we established an intuitive and systematic workflow capable of extracting and analyzing long-range coupled dynamics at the pMHC interface. The presented strategies were inspired by established analyses of MD simulations^107^. In the literature, correlational analyses of protein dynamics typically assess the pairwise cross-correlation of atomic displacement vectors, sometimes only of the Cα atoms, over single MD trajectories to identify concerted motions^86,108–110^. Instead, we analyzed the correlations in the magnitudes of the per-residue RMSF values over multiple paired trajectories. We were interested in identifying correlated differences in RMSF profiles after introducing a dynamic perturbation in the form of a p3P mutation, instead of the subtly different exercise of identifying concerted motions of atoms along a trajectory. The abstraction of protein dynamics on a per-residue basis was initially applied to address the challenge of appropriately aligning atomic indexes from different trajectories despite the presence of p3V/P mutations and, to a lesser extent, unmodeled termini. Subsequently, the use of fluctuation magnitude, rather than displacement vectors, was incorporated to reduce the risk of signal interference due to partially independent motions of the backbone and sidechain moieties of each residue^111^. It would prove interesting to combine our approach with more conventional dynamic cross-correlation analyses if we develop a strategy to systematically and reasonably address the misalignment of atomic indexes. Importantly, we demonstrate that a comparison of per-residue RMSF values correlates with immunogenicity (Supplementary Figs. 8, 15), particularly around residues confirmed to be both structurally and biochemically relevant^22,44,62,71–73,104,105^.

In terms of dynamical network construction, a range of different methods have been published^86,112,113^, including those that use correlation or distance thresholds. To our knowledge, only one other study has explored pMHC interface dynamics through a combination of both MD and network analyses^86^, however, significant differences exist between our methods. For instance, they utilize more conventional cross-correlation and suboptimal path analyses on a wide range of human HLA-A2 pMHCs to assess long-range allostery between the α3 domain and the peptide-binding cleft. We did not include the α3 domain in the downstream analyses of our MD trajectories, as we could not ensure the equilibration of interdomain motions between the α3 domain or β_2_m chain and the rest of the pMHC^96^. This provided the additional benefit of avoiding conclusions drawn from regions that could not be readily modeled crystallographically^62^. We further incorporated correlational significance testing and an additional optimization step with custom validation metrics to ensure our network was biophysically relevant. In the end, the network excelled at uncovering previously hidden dynamic couplings a the pMHC interface, which were not readily accessible through B-factor, ensemble refinement, or conventional MD trajectory analyses.

Regarding the formulation of the network edges, this work utilized Spearman rank correlation to identify monotonic relationships between the per-residue RMSF magnitudes. This approach, in conjunction with the available structural data^62^, supports the hypothesis that the p6F sidechain dynamics could be affected by p3P-induced altered dynamics of the α2-helix. Potential future approaches could explore different edge definitions. For example, mutual-information-based measures could further identify stochastic dependencies between nodes that are not monotonic, as has been done for other protein systems^114,115^.

Furthermore, the presented current workflow could be extended to include the TCR and assess the dynamic coupling of the p3P substitution or more conventional APL mutations at anchor positions with residues in the CDR loops^94^. If such relationships exist, they may be linked to important biophysical features that govern immunogenicity, such as the propensity for catch bond formation at the TCR interface^48–52^. Additionally, the role of coupled dynamics in stabilizing pMHC through β_2_m^93,105]^ could be investigated using similar methods. Identifying conserved networks of dynamically coupled residues across various pMHCs or pMHC-TCR pairings could significantly enhance our understanding of the co-evolution and germline-encoded affinity between pMHCs and TCRs^116^. Ultimately, this workflow, coupled with pMHC structure prediction pipelines^117^, may also be useful in pre-screening strategies and assessing the dynamics of tumor-associated antigens and neoantigens, as well as potential APLs for peptide vaccinations and other immunotherapeutic strategies^5^.

To conclude, we have established methods to systematically encode the correlated dynamics of the pMHC as a biophysically relevant network. The observations obtained from these methods align well with existing structural, biophysical, and functional data. In our highly characterized model system, this workflow is still capable of identifying a novel dynamic hypothesis by which the altered dynamics at p3V/P are propagated through the α2-helix to influence the conformational sidechain sampling of p6F. As such, the network-based representation of residue dynamics could facilitate a systematic dynamic understanding of differential TCR-pMHC immunogenicity.

## Methods

### Atomic coordinates and ensemble refinement of complexes

Fully refined models for all pMHC and TCR/pMHC complexes were obtained from the following Protein Data Bank (PDB) entries: 1S7U, 4NSK, 1S7X, 3TBY, 5TJE, 5TIL, and 5M02^62,71,73^. Other similar complexes, such as H-2D^b^/Y4A/P14 (5M00), H-2D^b^/PA/P14 (5M01), H-2D^b^/Y4A (3QUK), H-2D^b^/PA (3TBS), H-2D^b^/Y4S (3QUL), and H-2D^b^/PS (3TBT), were excluded as they have hitherto not been characterized to the same extent as the peptides in this study. Furthermore, Y4S and Y4A were APLs designed to explore the mechanisms of T cell agonism^118,119^. It therefore seemed reasonable to restrict our study to the immunodominant epitope gp33, a common escape variant Y4F, and their respective proline-APLS. In *Coot*^120^, alternative conformations with lower occupancy were removed, and the model was re-refined using *phenix.refine*^121,122^ from the PHENIX 1.19.1-4122 program suite^123^. The models and phases from the refinement were used as input for the ensemble refinement. The ensemble refinement was performed according to previously published work^95,124,125^. *phenix.ensemble_refinement* generates the ensembles via a maximum-likelihood time-averaged restrained molecular dynamics simulation; default parameters were used except for the pTLS parameters, which were optimized for each refinement with values of 1.0, 0.9, 0.8, and 0.7.

### Structural and ensemble analysis and graphics

The per-residue root-mean-square fluctuations (RMSF) of every ensemble model were obtained using the *ens_tools.py* PyMOL script included with *phenix.ensemble_refinement*^124^. Structural analyses and representations were performed using PyMOL.

### MD simulations of the H-2D^b^/peptide complexes

All simulations were performed based on previously published protocols^106,126^ using GROMACS 2021.4^127^. The initial configurations were obtained from each individual pMHC molecule in the asymmetric unit cell of the following PDB entries: 1S7U, 4NSK, 1S7X, and 3TBY. Gaps and missing sidechains were modeled in *Coot* with the help of the deposited electron density maps, and, if required, the first molecule in the H-2D^b^/gp33 crystal structure as reference. Missing termini were not modeled. To account for the bias introduced by our choice of initial configuration, p3 was mutated manually in *Coot* to valine or proline as required, to generate the initial configurations for another set of MD simulations. Real-space refinement in *Coot* was used to remove model distortions in p3 and the immediately preceding and succeeding residues. Using the Amber99SB-ILDN forcefield^128^ and SPC/E water model^129^, the topology was generated through the *gmx pdb2gmx* command passed with the *-heavyh* flag. The latter allows for the use of 5 fs timesteps in MD runs through the hydrogen mass repartitioning scheme^130^ when constraining all bonds with the LINCS algorithm^131^. Each system was solvated in 150 mM NaCl in a cubic box with periodic boundary conditions and a minimum solute-to-box distance of 1.1 nm. Charges were neutralized by adding Na^+^ and Cl^-^ counterions. Following steepest-descent energy-minimization, each system was equilibrated, first a 200 ps NVT simulation with protein position restraints, then a 1 ns NPT simulation. Boltzmann-distributed velocities were assigned at the beginning of the NVT equilibration. Short-range van der Waals and Coulomb interactions were implemented with a 1.0 nm cutoff, using potential shifting to minimize cutoff truncation artifacts. Long-range electrostatics were handled with the Particle-Mesh Ewald method^132^. Long-range dispersion corrections for energy and pressure were also applied. Two thermostats, one for the solvent and one for the solute, were implemented through the velocity scaling algorithm^133^. For the NPT equilibration, pressure was controlled with a Parrinello-Rahman barostat^134,135^. After equilibration, the system was simulated for 200 ns using the same parameters as the NPT equilibration with system coordinates being saved every 100 ps. An interval of 100 ps was chosen because the resulting RMSF profiles were in close agreement with those derived from 10-ps-interval simulations. Additionally, the rotameric distributions of more dynamic residues, such as p6F, were similar at 100 and 10 ps intervals. This resulted in 26 trajectories, one for each crystal copy and each in silico-mutated copy. Each trajectory was gathered and centered on the H-2D^b^/peptide complex and each frame was roto-translationally fitted to the backbone of the first frame. All simulations were validated by monitoring the root-mean-square deviations compared to the input structure, radius of gyration, and visual inspection of each trajectory for large, unexplained divergences. Additionally, the temperature, pressure, density, and energies were confirmed to be constant.

### Code environments for dynamic analysis of MD trajectories

R and Python 3 were used to perform analyses on the MD data. The R analyses were run in an R 4.4.1^136^ environment with the readxl 1.4.3^137^, foreach 1.5.2^138^, doParallel 1.0.17^139^, tidyverse 2.0.0^140^, and ggplot2 3.5.1^141^ packages installed. All Python 3 analyses were run using JupyterLab 4.0.6 notebooks^142^ in a Conda 24.9.1 environment (Anaconda) with Python 3.10.12 (Python Software Foundation) and the NumPy 1.26.0^143^, SciPy 1.13.1^144^, pandas 2.1.1^145^, Matplotlib 3.8.0^146^, scikit-learn 1.5.1^147^, NetworkX 3.3^148^, Biopython 1.81 ^149^

, MDTraj 1.10.0^150^, and Multiprocess 0.70.15^151^ libraries installed.

### Trajectory analysis and graphics

The per-residue RMSF values were obtained from each trajectory using the energy-minimized structure as reference through the *gmx rmsf* command with the *-res* flag. This RMSF data was subsequently analyzed and plotted using custom R scripts. To visually compare the dynamics across different trajectories, the *gmx rmsf* command was used again to convert the atomic RMSF to anisotropic B-factors, which were written to the B-factor column of a PDB file with the energy-minimized coordinates. This was visualized in PyMOL using stick and ellipsoid representations. The backbone and dihedral angle distributions of each peptide residue throughout all trajectories were obtained using MDTraj.

### Correlational analysis of the RMSF data obtained from MD simulations

The Spearman rank correlation coefficient (*ρ*_*s*_) of the per-residue RMSF data was calculated for all pairwise combinations of residues to assess their monotonic relationship. Subsequently, the coefficients were converted to the following test statistic (*t*), where *n* denotes the number of simulations:

$$t={\rho }_{s}\sqrt{\frac{n-2}{1-{{\rho }_{s}}^{2}}}$$

To assess the significance of the observed correlation coefficients, the corresponding p-values were obtained through a two-tailed comparison of the test statistics against the null distribution. This null distribution was generated from test statistics calculated from the correlation of 5 x 10^7^ pseudo-randomly paired resamples of two sequential integer arrays from 1 to 26 inclusive. The null distribution was obtained using the *scipy.stats.permutation_test* function and the two-tailed comparison was performed with the nested functions of *scipy.stats.permutation_test*^144,152^. These nested functions were copied to our script to avoid having to compute the null distribution repeatedly. Multiple testing corrections were applied by adjusting the p-values using the Benjamini-Hochberg procedure to control the estimated false discovery rate at 5% significance^153^.

As an alternative approach, hierarchical agglomerative clustering was applied to the per-residue RMSF data. The clustering was achieved with the UPGMA algorithm^154^ using the pairwise correlation distance of the ranked RMSF data, equivalent to $1\,-\,{\rho }_{s}$, as the distance metric. The threshold for clustering leaves on the dendrogram was determined by identifying a cophenetic distance threshold which included p3V/P in one of the clusters.

### Correlational analysis of per-residue RMSF data and immunogenicity

The per-residue RMSF values were grouped according to immunogenicity, where p3P trajectories were considered to be more immunogenic than p3V trajectories. This aligns with the published biophysical and functional data^62^. For each residue of interest, the rank biserial correlation of RMSF values with immunogenicity was calculated. Subsequently, residues with the largest magnitudes of correlation were further subjected to permutation testing to determine the probability of observing the same result through random chance. The permutation test was performed using the same test statistic as before, however, due to the small number of comparisons, no generalized null distribution was generated. Instead, a null distribution was generated from 1 x 10^5^ pseudo-random resamples of the paired RMSF and immunogenicity data for each residue tested.

### Dynamic correlation network construction and validation

Undirected networks with weighted edges were constructed from the correlation matrix of per-residue RMSF data. In these networks (*G*), all interface residues were assigned as nodes (*V*) and edges and their respective weights (*E*) were derived from the correlation matrix:

$$G=(V,E)$$

A plausible general edge weight metric was defined to later optimize the construction of a biophysically relevant network:

$$E\,\left[u,v,n,t\right]=\left\{\begin{array}{l}\begin{array}{l}\frac{\left|{\rho }_{s}\left[u,v\right]\right|}{{\left({r}_{C\alpha }\left[u,v\right]\right)}^{n}},{r}_{C\alpha }\left[u,v\right] < t\wedge {p}_{{adj}}\left[u,v\right] < 0.05\\ {\rm{\theta }},\mathrm{otherwise}\end{array}\end{array}\right.$$

*u* and *v* are any two nodes in the network (*u, v ∈ V*), *ρ*_*s*_ is their Spearman rank correlation, *r*_*Cα*_ is their through-space C_α_ distance derived from the crystal structure of H-2D^b^/gp33, and *p*_*adj*_ is their Benjamini-Hochberg corrected p-value. Both *n* and *t* are parameters to be optimized and represent the inverse power of distance and distance threshold respectively.

A 2D scan of possible *n* and *t* values was performed to generate different networks. Custom shortest-path-based metrics were defined to help validate each network and assess their biophysical relevance to probing network dynamics. All shortest paths (*P*) for the network *G* were calculated using Dijkstra’s algorithm^155^:

$$\forall P\in G,P=\left({v}_{\mathrm{start}},{v}_{1},\ldots ,{v}_{n},{v}_{\mathrm{end}}\right)$$

These shortest paths were used to calculate the proportion of shortest paths with any through-space edge distance greater than the initial distance (*F*_*1*_), the proportion of shortest paths where the through-space distance increases relative to the preceding nodes (*F*_*2*_), and the proportion of shortest paths where the through-space distance increases relative to the initial distance (*F*_*3*_):

$${F}_{1}\left[G\right]=\frac{{\sum }_{P\in G}\,{f}_{1}\left[P\right]}{{\sum }_{P\in G}\,},{\mathrm{where}}\,{f}_{1}\left[P\right]=\left\{\begin{array}{l}\begin{array}{l}1,{r}_{C\alpha }\left[{v}_{n},{v}_{n+1}\right] > {r}_{C\alpha }\left[{v}_{{\mathrm{start}}},{v}_{{\mathrm{end}}}\right]\\ \,\,\,\,\,\,\,\,\,\,\,\,\,\,\,\,\,\,\,0,{\mathrm{otherwise}}\end{array}\end{array}\right.$$

$${F}_{2}\left[G\right]=\frac{{\sum }_{P\in G}\,{f}_{2}\left[P\right]}{{\sum }_{P\in G}\,},{\mathrm{where}}\,{f}_{2}\left[P\right]=\left\{\begin{array}{l}\begin{array}{l}1,{r}_{C\alpha }\left[{v}_{n},{v}_{n-1}\right] < {r}_{C\alpha }\left[{v}_{n},{v}_{n+1}\right]\\ \,\,\,\,\,\,\,\,\,\,\,\,\,\,\,\,\,0,\mathrm{otherwise}\end{array}\end{array}\right.$$

$${F}_{3}\left[G\right]=\frac{{\sum }_{P\in G}\,{f}_{3}\left[P\right]}{{\sum }_{P\in G}\,},{\mathrm{where}}\,{f}_{3}\left[P\right]=\left\{\begin{array}{l}\begin{array}{l}1,{r}_{C\alpha }\left[{v}_{n},{v}_{{\mathrm{end}}}\right] > {r}_{C\alpha }\left[{v}_{{\mathrm{start}}},{v}_{{\mathrm{end}}}\right]\\ \,\,\,\,\,\,\,\,\,\,\,\,\,\,\,\,\,\,\,\,\,0,{\mathrm{otherwise}}\end{array}\end{array}\right.$$

In the end, a biophysically relevant network was chosen based on a compromise between minimizing *F*_*1*_ and edge weight complexity, while maximizing the number of nodes present in the largest connected component (> 90%). *F*_*2*_ and *F*_*3*_ were not directly used in network optimization but are, nonetheless, informative for understanding network properties.

### Network analysis

Our chosen biophysically relevant (*n* = 0, *t* = 10 Å) and significant correlation network (*n* = 0, *t* = 52 Å) were subject to a suit of standard analyses to investigate network features. Note that the largest observed pairwise C_α_ distance was 51.1 Å. The topological properties of the networks, such as node centrality, were assessed using built-in functions from NetworkX and mapping these values back onto the crystallographic structure of H-2D^b^/gp33. For all centrality measures, except for degree centrality and coreness, edge weight (*E*) or edge distance (*E*^*-1*^) were incorporated as required. The shortest paths originating from p3V/P, as identified previously using Dijkstra’s algorithm, were also mapped to the structure. Lastly, the community structure of dynamics at the pMHC interface was also assessed using the Louvain community detection algorithm^156^. Due to the heuristic nature of this approach, the Louvain algorithm was iteratively performed on the network with 1 x 10^6^ pseudo-random seeds. Both the most frequent solution and community co-occurrence frequency of each residue with p3V/P were mapped to H-2D^b^/gp33.

## Supplementary information

41540_2026_653_MOESM1_ESM.

## Data Availability

All MD data and code generated and analyzed during the current study are available from the following Zenodo repository: 10.5281/zenodo.14265476.
