# Supplementary material for: Dynamical network analysis reveals long-range residue couplings at the pMHC interface underlying enhanced immunogenicity
Source: NPJ Syst Biol Appl. 2026 Jan 28;12:15. doi: 10.1038/s41540-026-00653-y (PMC12855856; doi:10.1038/s41540-026-00653-y)
Supplement: Supplementary file 1 — 41540_2026_653_MOESM1_ESM. [file 41540_2026_653_MOESM1_ESM.pdf]

Supplementary Table 1. Ensemble refinement statistics for each crystal structure. [a] ΔR_{free} was calculated by subtracting the ensemble refinement ΔR_{free} from the published ΔR_{free}. Multiple conformations were removed from H-2D[b]/PF/P14 before ensemble refinement.

Complex	PDB code	Space group	Resolution	R_{free}		ΔR_{free}[a]	Ensemble size	Nr. AU molecules
				PDB	Ensemble			
H-2D[b]/gp33	1S7U	$P\ 1\ 2_1\ 1$	2.20 Å	0.262	0.280	0.018	27	4
H-2D[b]/V3P	4NSK	C 1 2 1	2.60 Å	0.283	0.267	-0.016	30	1
H-2D[b]/Y4F	1S7X	$P\ 1\ 2_1\ 1$	2.41 Å	0.254	0.246	-0.008	24	4
H-2D[b]/PF	3TBY	$P\ 1\ 2_1\ 1$	2.50 Å	0.315	0.311	-0.004	18	4
H-2D[b]/gp33/P14	5TJE	$P\ 2_1\ 2_1\ 2_1$	3.20 Å	0.314	0.334	0.020	14	2
H-2D[b]/V3P/P14	5TIL	$P\ 2_1\ 2_1\ 2_1$	2.83 Å	0.275	0.295	0.020	14	2
H-2D[b]/PF/P14	5M02	C 1 2 1	1.75 Å	0.214	0.194	-0.020	47	1

Supplementary Table 2. All polar contacts of each crystal copy in the TCR-unbound pMHCs.

All residues with favorable or unfavorable polar contacts within 4 Å are indicated.

Complex	Copy	Chain	Polar contacts (<4.0 Å)
H-2D^b/gp33	A	H-2D^b	E31 E58 E61 R62 Q65 K68 G69 Q70 Y84 Y85 N86 Q87 S88 A89 G90 G91 S92 H93 D106 R108 E119 R121 D137 M138 K186 T214 P267
		β2m	N42 E74 T75 T77 E89 D96
		gp33	p4Y p5N
	B	H-2D^b	Q54 E61 R62 Q65 G69 Q70 Y84 Y85 N86 Q87 S88 A89 G90 G91 S92 H93 R108 E119 R121 E137 M138 K173 R194 K196 E223 Y262 P267
		β2m	N42 K44 E74 T75 T77 E89 P90 D96
		gp33	p4Y p5N
	C	H-2D^b	R62 R79 Y84 Y85 N86 Q87 S88 A89 G90 G91 S92 W107 R108 E119 R121 D137 M138 K146 S150 N174 K186 R194 S195 E223 E264
		β2m	Q2 K19 N42 E74 T75 P90 T92 Y94 W95 D96
		gp33	p4Y p8T
	D	H-2D^b	G16 L17 E18 E53 Q54 R62 R79 Y84 Y85 N86 Q87 S88 A89 G90 G91 S92 H93 W107 R108 E119 R121 D137 M138 A139 K146 Q149 N176 K186 E223 E268
		β2m	Q2 N42 E74 T75 T92 Y94 W95
		gp33	p4Y p8T
H-2D^b/V3P	A	H-2D^b	Q54 E58 Q65 R79 N80 N86 S88 G90 G91 R108 R121 A136 D137 M138 Q141 R144 K146 Q149 S150 H155 G162 E166 R170 K173 N220 E222 P267 E268 H269 R273
		β2m	Q2 N42 T75 D76 D85 S86 M87 A88 E89 W95
		V3P	p4Y p8T
H-2D^b/Y4F	A	H-2D^b	E41 E58 Q65 G69 Y84 Y85 N86 Q87 S88 A89 G90 G91 S92 H93 D106 W107 E119 R121 D137 M138 K173 R194 E223 P267
		β2m	N42 E74 T75 T77
		Y4F	p5N
	B	H-2D^b	E41 R62 G69 Q70 Y84 Y85 N86 Q87 S88 A89 G90 G91 H93 E119 R121 D137 M138 E166 R194 S195 K196 E223 Y262 P267 E268
		β2m	N42 E74 T75 T77 D96 R97
		Y4F	p5N
	C	H-2D^b	G16 E18 Q54 R75 R79 N80 Y84 Y85 N86 Q87 S88 A89 G90 G91 S92 H93 W107 R108 E119 R121 A136 D137 M138 A139 K146 K173 K186 S195 E222
		β2m	Q2 K19 G43 E74 T75
		Y4F	p8T
	D	H-2D^b	E18 E53 Y84 Y85 N86 Q87 S88 A89 G90 G91 S92 H93 W107 R108 E119 R121 D137 M128 A136 K146 S150 N174 T178 S195 H263 P267 E268
		β2m	H13 K19 E74 T75 W85 T92
		Y4F	p8T
H-2D^b/PF	A	H-2D^b	E58 R62 Q65 K68 G69 Q70 Q72 Y84 Y85 N86 Q87 S88 G90 G91 S92 H93 W107 E119 R121 D137 M138 N176 R181 R194 T214 P267
		β2m	E16 N42 T75 T77 E89 K91 T92
		PF	p5N
	B	H-2D^b	R62 Q65 G69 Q70 Q72 Y84 Y85 N86 Q87 S88 G90 G91 S92 H93 W107 E119 R121 D137 M138 T178 R181 R194 E223 H263 P267
		β2m	E16 N42 E74 T77 E89 K91 T92 Y94 W95
		PF	p5N
	C	H-2D^b	E18 R62 Q72 R79 Y84 Y85 N86 Q87 S88 G90 G91 S92 H93 S105 W107 R108 E119 R121 D137 M138 K146 N176 R181 K186 P267
		β2m	N17 E74 T75 E89 Y94 R97
		PF	p8T
	D	H-2D^b	E18 R62 Q72 Y84 Y85 N86 Q87 S88 G90 G91 S92 H93 W107 R108 E119 R121 D137 M138 K146 S150 G175 R181 K186 P267
		β2m	Q2 N17 T73 E74 T75 S86 E89 Y94 W95 D96 R97
		PF	p8T

Supplementary Table 3. All polar contacts of each crystal copy in the TCR-bound pMHCs.

All residues with favorable or unfavorable polar contacts within 4 Å are indicated.

Complex	Copy	Chain	Polar contacts (<4.0 Å)
H-2D^{b}/gp33/P14	A	TCRα	S17 Q16 T19 T25 V60 D62 K117
		TCRβ	H39 T53 K121 V122 L124 E126 K175 N178 Y179 R198 N227 S229 E231 A230
		H-2D^{b}	L17 E18 N86 K103 D106 W107 R145 H169 T214 N220 G221 E264 L266 P267 E268 R273
		β2m	K19 E36 Q38 N42 G43 K44 K45 R81 Y94
		gp33	–
	B	TCRα	Q1 K3 E4 K5 V60 D62 R76 K117
		TCRβ	D36 H39 R42 D51 T53 E54 K175 E176 S177 N178 Y181 V122 L124 N159 G160 R198 H200 Q226 R235 D237
		H-2D^{b}	L17 E18 D106 W107 R144 E148 K173 T182 D183 T214 N220 G221 L266 P267 E268
		β2m	P20 N21 E36 Q38 K44 R81
		gp33	–
H-2D^{b}/V3P/P14	A	TCRα	Q16 S17 T19 T25 V60 S61 D62 K117
		TCRβ	D36 T37 Y48 D51 K63 K175 N178 Y179 K121 V122 L124 E126 R198 N227 I228 S229 E231 R235
		H-2D^{b}	L17 E18 Y84 Y85 N86 D106 W107 R108 R145 K173 T214 N220 G221 E223 L266 P267
		β2m	K19 E36 Q38 K44 K45 T75 D76 R81 Y94
		V3P	–
	B	TCRα	Q2 E4 H6 D7 V60 D62
		TCRβ	D36 R42 D51 T53 E54 K63 V122 S123 L124 E126 N159 G160 Y174 K175 E176 N178 R198 R202 Q226 D237
		H-2D^{b}	L17 E18 D106 W107 R108 R144 E148 K173 R181 T182 T214 N220 G221 E223 L266 P267
		β2m	H13 P20 N21 E36 Q38 K44 K45 T75 R81 Y94
		V3P	–
H-2D^{b}/PF/P14	A	TCRα	V60 S61 D62 K63 R134 Q136 T155
		TCRβ	T13 D51 T53 E54 E112 D113 R115 K129 N134 N158 N178 Y179 R198 E218 R202 K222 Q226 S229 A236
		H-2D^{b}	L17 E18 E53 Q54 E58 D106 W107 R108 K131 A135 A136 D137 M138 Q141 R145 H169 K157 K173 H191 G197 T214 N220 G221 E222 E223 Q255 N256 Y262 L266 P267
		β2m	E36 Q38 N42 K44 K45 E74 R81 Y94
		PF	–

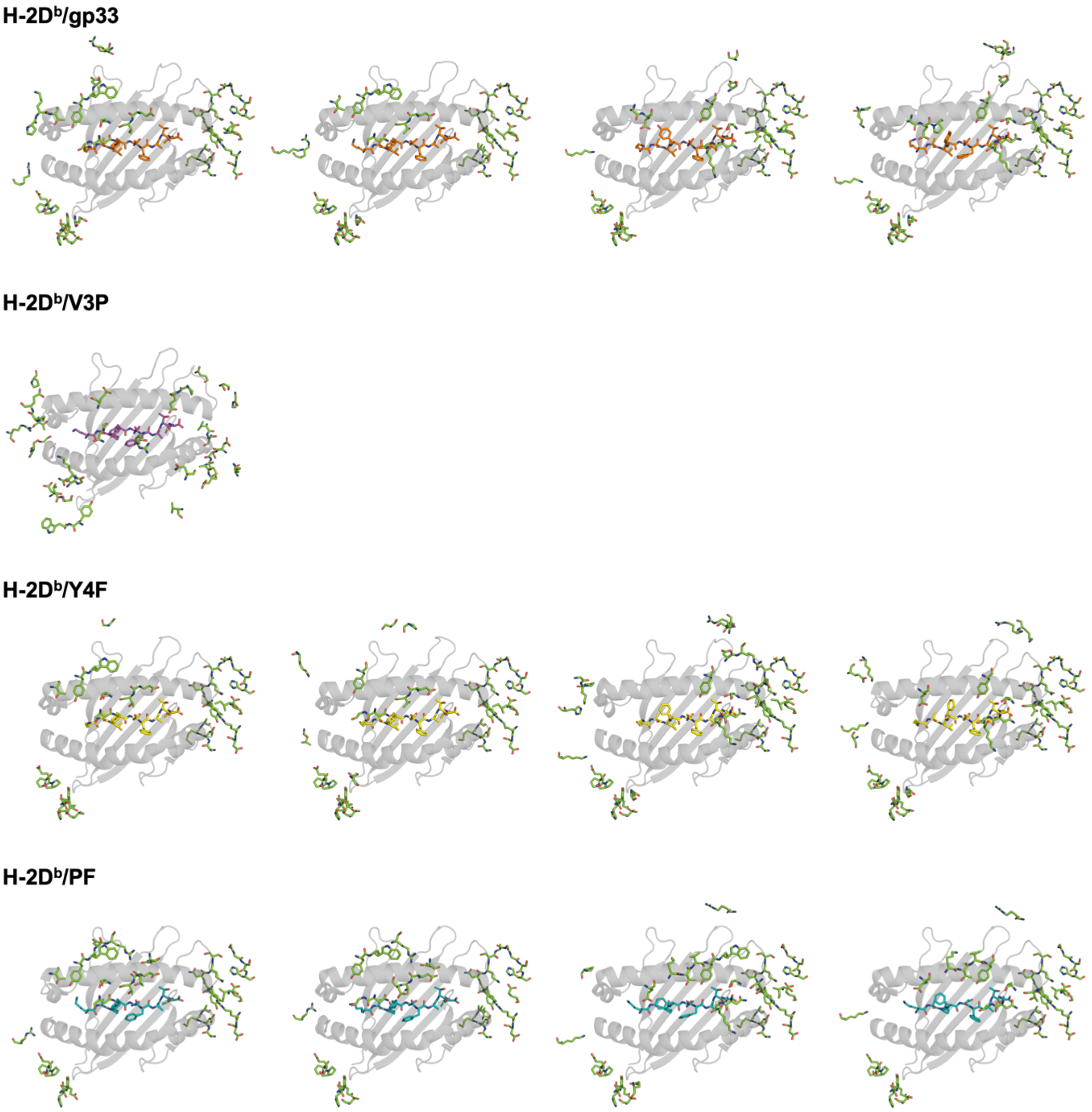

Supplementary Fig. 1. The crystal packing geometry at the pMHC interface is similar for H-2D^b/gp33, H-2D^b/Y4F, and H-2D^b/PF. The α1 and α2 domains are shown as a transparent gray cartoon. Each peptide is shown in stick representation, with gp33 (orange), V3P (purple), Y4F (yellow), and PF (teal) color accordingly. Any residues not part of the respective crystal copy within 4 Å (green) are in stick representation.

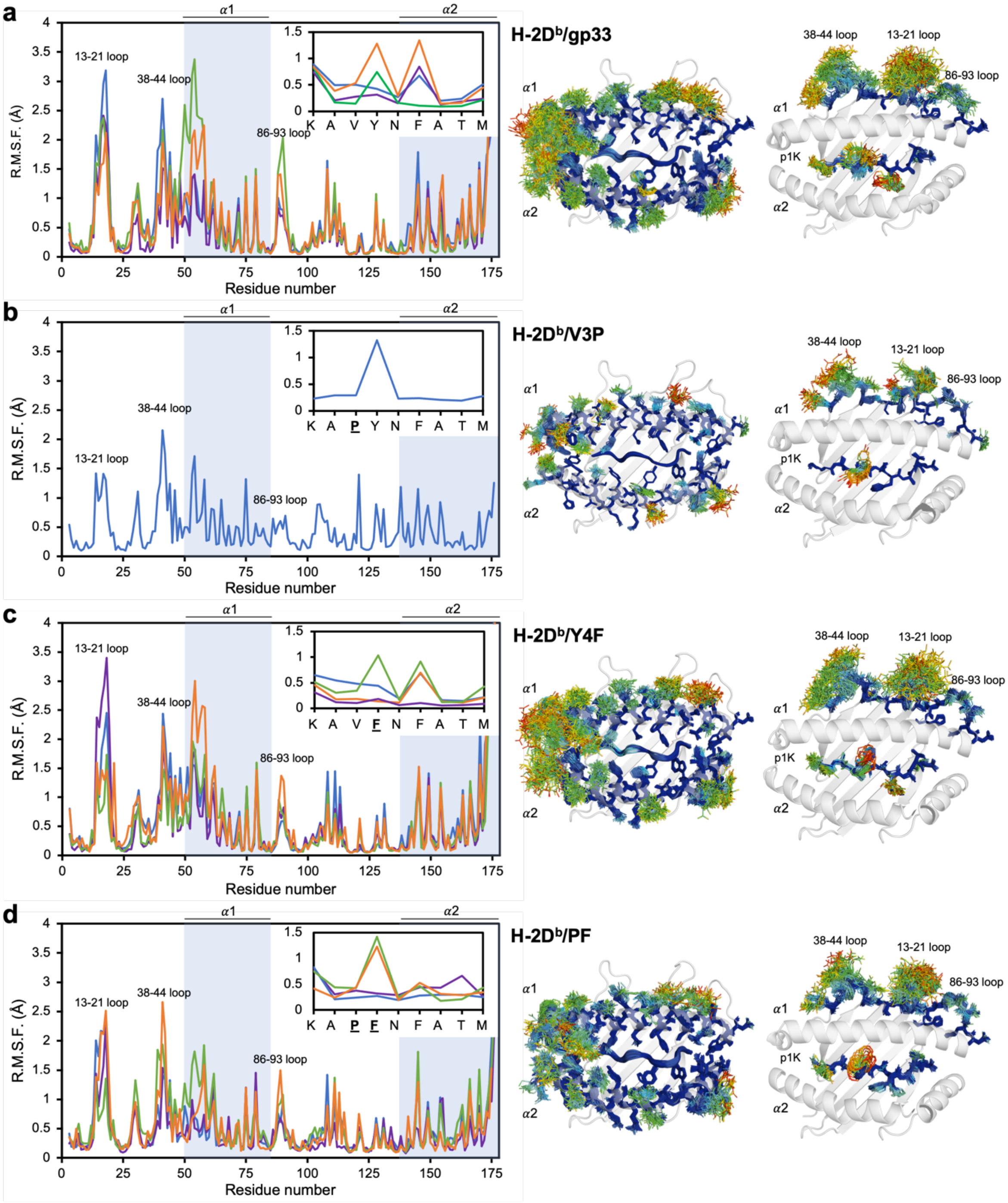

Supplementary Fig. 2. MHC and peptide conformational flexibility are reduced when proline is positioned at p3. The per-residue RMSF values, obtained from ensemble refinement, are reported for residues 1 to 176 of MHC and all peptide residues (inset). MHC helixes and most dynamic loop regions are labeled. Data are reported using different line colors for every molecule

in the asymmetric unit (blue for copy A, purple for copy B, green for copy C and orange for copy D). Ensemble structural regions are represented as sticks and colored by RMSF, ranging from 0 (deep blue) to 4 (bright red) Å. pMHC structures represent the complex before P14 binding. **a** A high degree of mobility is seen in H-2D^b/gp33, particularly at the beginning of the α1 helix, and by the loops, including residues 13-21 and 38-44. **b** The pMHC fluctuations are drastically reduced in H-2D^b/V3P. The only residue of the peptide exploring multiple conformations is p4Y. **c** A similar degree of dynamics is observed in H-2D^b/Y4F compared to H-2D^b/gp33. However, p4F and p6F display reduced flexibility. **d** The dynamics of the pMHC are reduced in H-2D^b/PF, despite p4F regaining mobility.

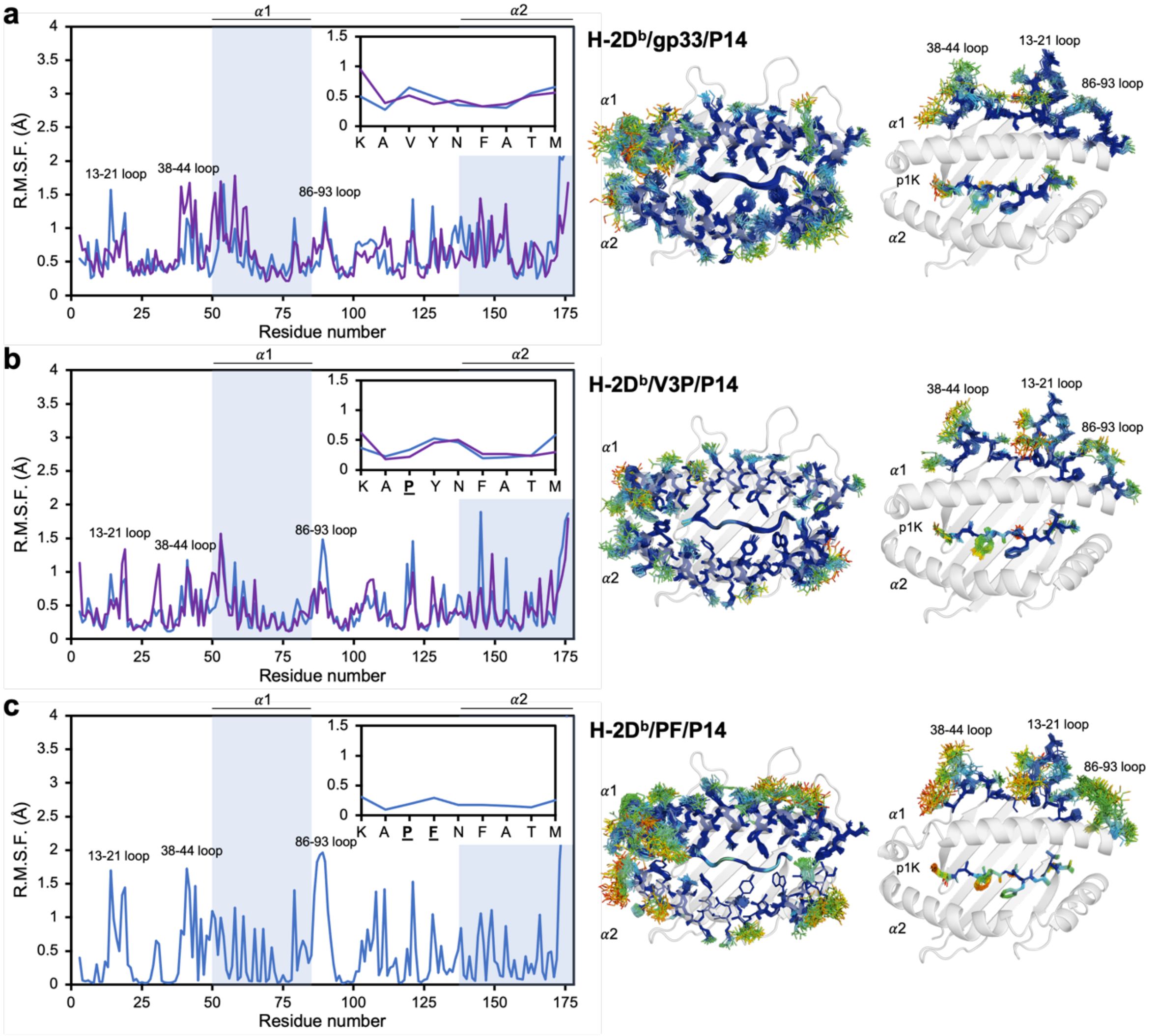

Supplementary Fig. 3. MHC and peptide conformational flexibility are reduced when the TCR P14 binds to the complexes. The per-residue RMSF values, obtained from ensemble refinement, are reported for residues 1 to 176 of MHC and all peptide residues (inset). MHC helixes and the most flexible and dynamic loop regions are labeled. Data are plotted with different line colors for every molecule in the asymmetric unit (blue for copy A, purple for copy B). Ensemble structural regions are represented as sticks and colored by RMSF, ranging from 0 (deep blue) to 4 (bright red) Å. All pMHC structures represent the complex after P14 binding. **a** Reduced dynamics are observed in H-2D^b/gp33 after P14 binding. **b** The fluctuations in the pMHC are

further reduced in H-2D^b/V3P. **c** Increased mobility is visible around the region of α1 helix in H-2D^b/PF, particularly in the loop containing residues 86-96. Overall, the pMHC complexes bound to P14 display reduced dynamics compared to the free pMHC complexes.

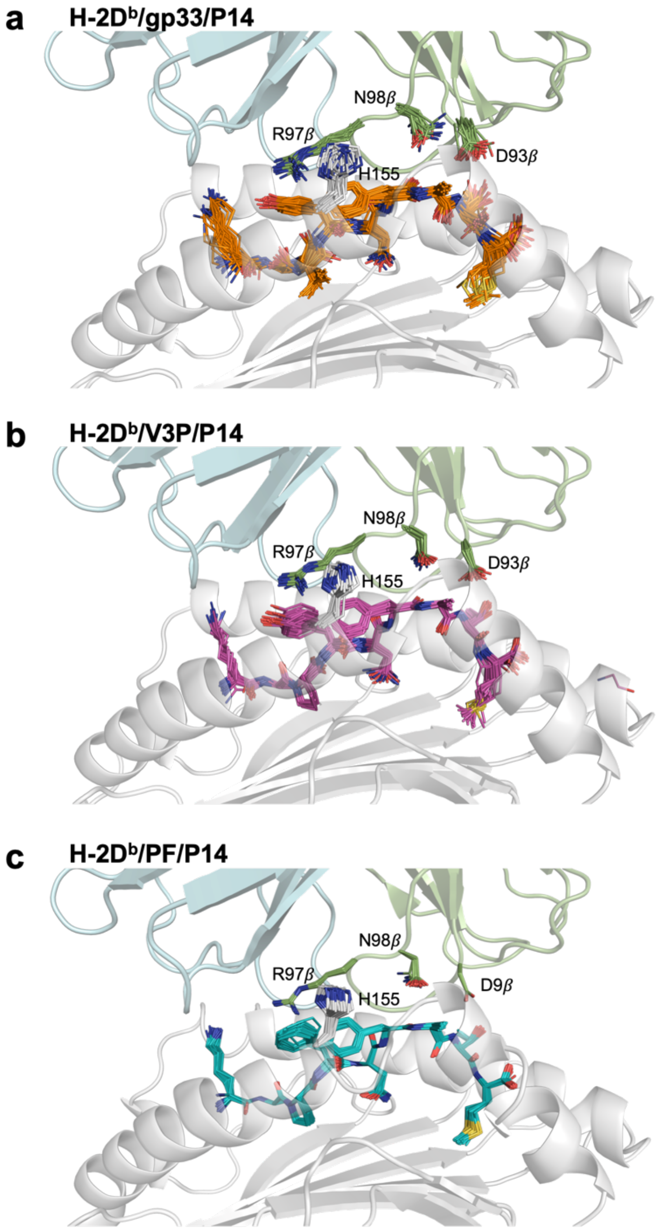

Supplementary Fig. 4. The TCR-peptide interface is rigid and no relationship is observable between pMHC-TCR affinity and dynamics in this region of the ternary complex. a, **b**, **c** Cartoon representation of the MHC heavy chain (grey) and P14 α (light blue) and β (green) chains. Peptide residues, a key interacting residue of the MHC (H155), and TCR (D93β, R97β, and N98β) are shown as sticks. The conformations of the labeled residues are similar in all ensembles, with H-2D^b/gp33/P14 displaying the greatest degree of fluctuations.

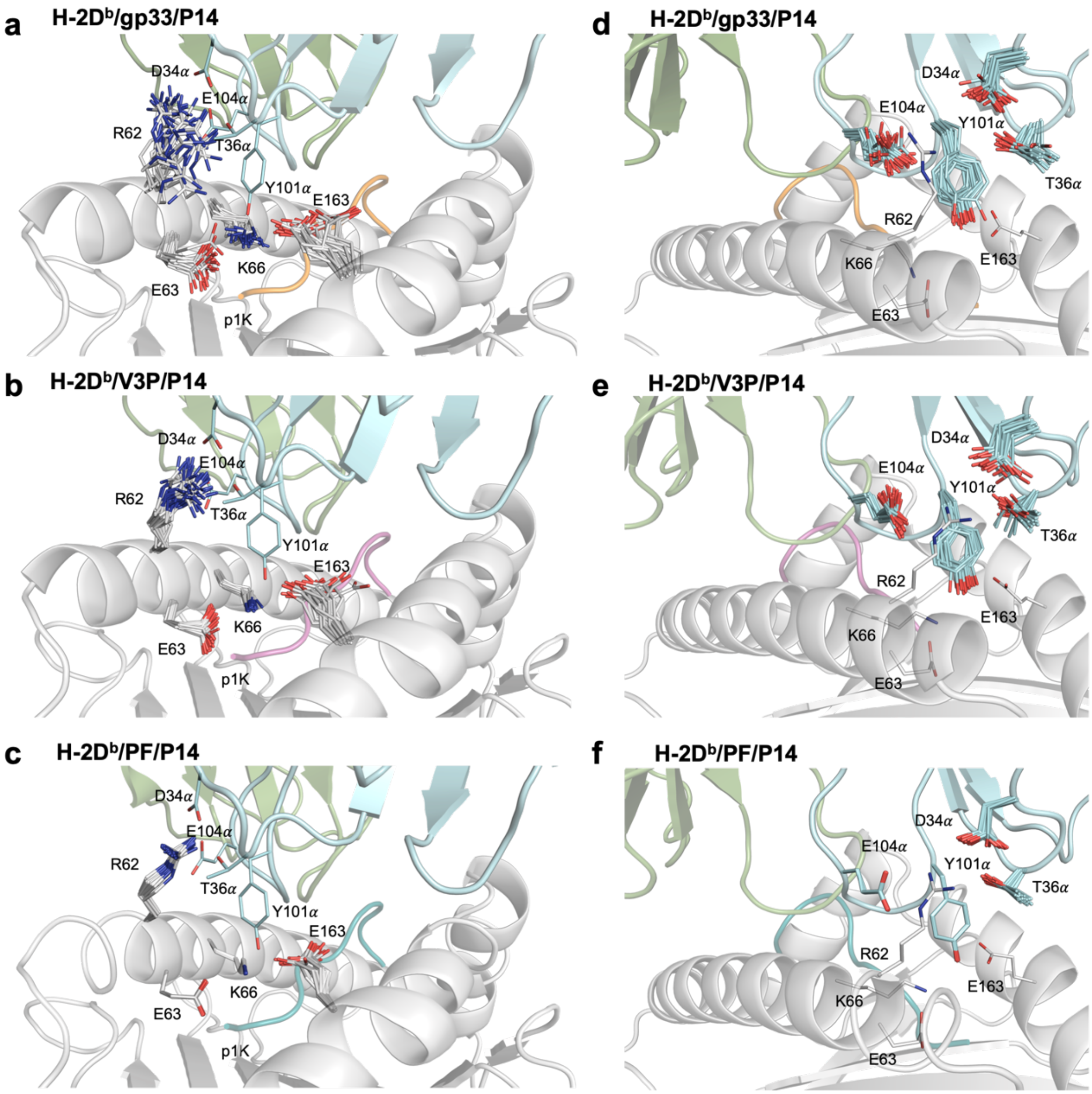

Supplementary Fig. 5. The conformational rigidity at the pMHC-TCR interface does not correlate with the affinity of the TCR-pMHC interactions. a, **b**, **c**, **d**, **e**, **f** Cartoon representation of the heavy chain (grey), peptides and P14 α (light blue) and β (green) chains. Key interacting residues of the MHC (R62, E63, K66, E163) and the TCR (D34α, T36α, Y101α, E104α) are shown as sticks. Sidechain ensembles of MHC heavy chain residues are shown in **a**, **b**, and **c**, while sidechain ensembles of TCR α chain residues are shown in **d**, **e**, and **f**. The conformation of the highlighted residues is similar in all ensembles of complexes, with H-2D^b/gp33/P14 and H-2D^b/V3P/P14 having R62 and E163 displaying higher conformational flexibility.

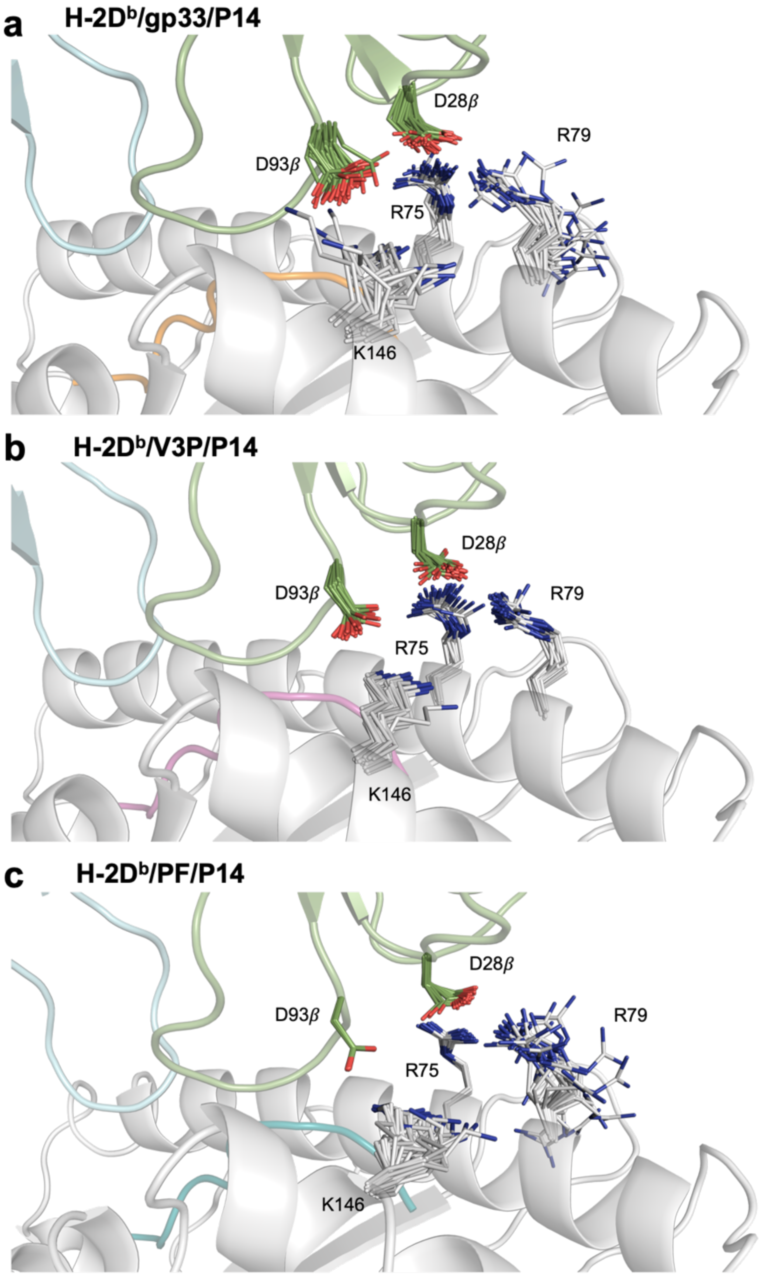

Supplementary Fig. 6. The conformational rigidity at the pMHC-TCR interface does not correlate with the affinity of the TCR-pMHC interactions. a, **b**, **c** Cartoon representation of the heavy chain (grey), peptides, and P14 α (light blue) and β (green) chains. The sidechains of the key interacting MHC (R75, R79, K146) and TCR (D93β, D28β) residues are shown as sticks. R79 and K146 sidechains in H-2D^b/gp33/P14 and H-2D^b/PF/P14 complexes display increased conformational flexibility compared to H-2D^b/V3P/P14.

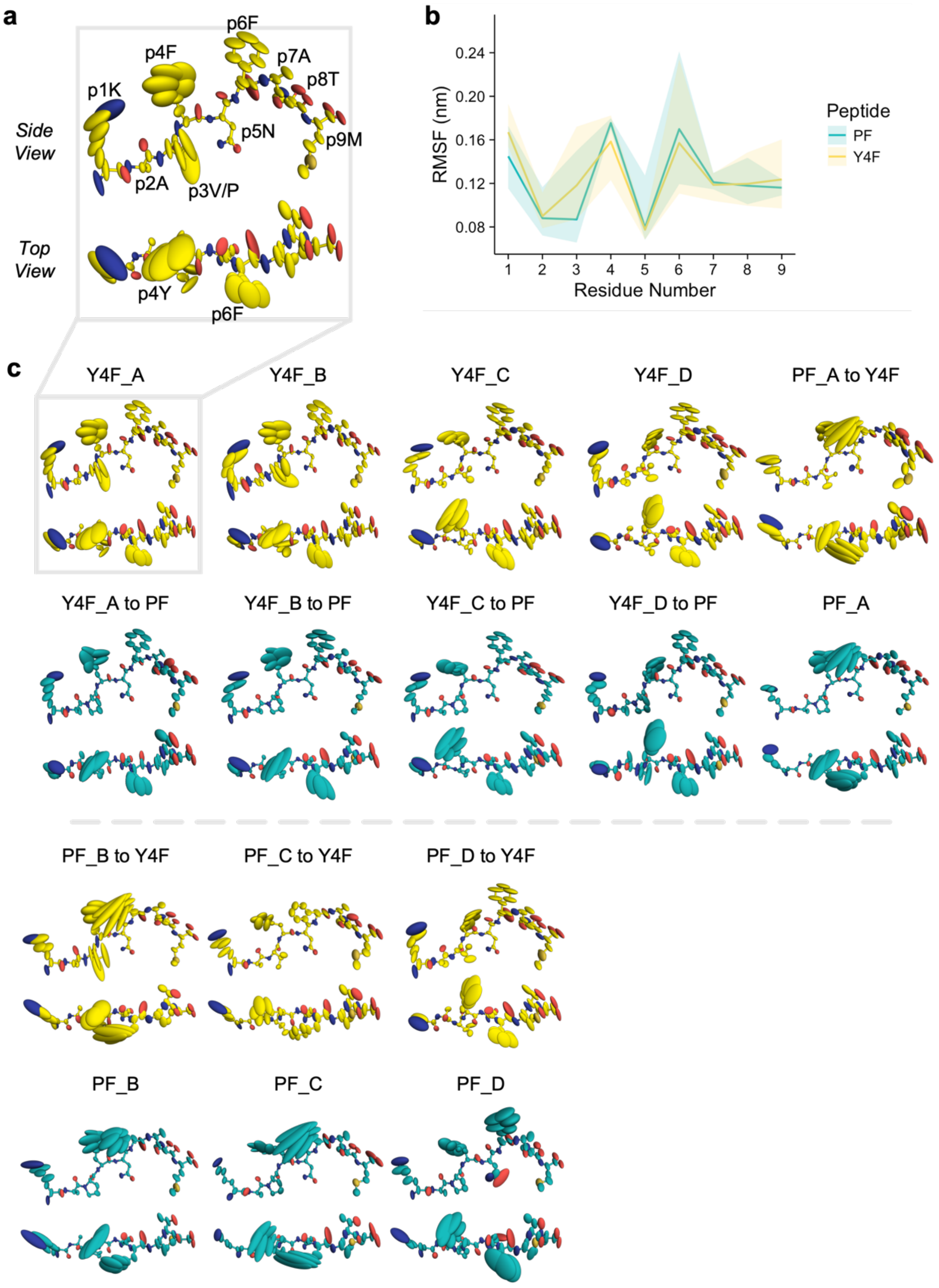

Supplementary Fig. 7. Overview of the conformational sampling of the Y4F and PF peptides throughout the MD trajectories reveals that the proline substitution leads to rigidification at p3 and increased dynamics at p6F. Stick and ellipsoid representation of MHC-bound Y4F

(yellow) and PF (purple). Ellipsoids are scaled and shaped according to the anisotropic atomic temperature factors derived from each trajectory and mapped to their respective crystallographic models. **a** Residue labeling of side and top views of the peptide as reference. **b** Average per-residue RMSF values over all trajectories for Y4F and PF. The colored transparent envelopes depict the full range of observed trajectory RMSF values for each peptide. **c** Conformational sampling of the peptides observed in each trajectory. Paired simulations are organized vertically, with one trajectory derived from the initial crystallographic model and the other derived from an *in silico* mutation between p3V and p3P.

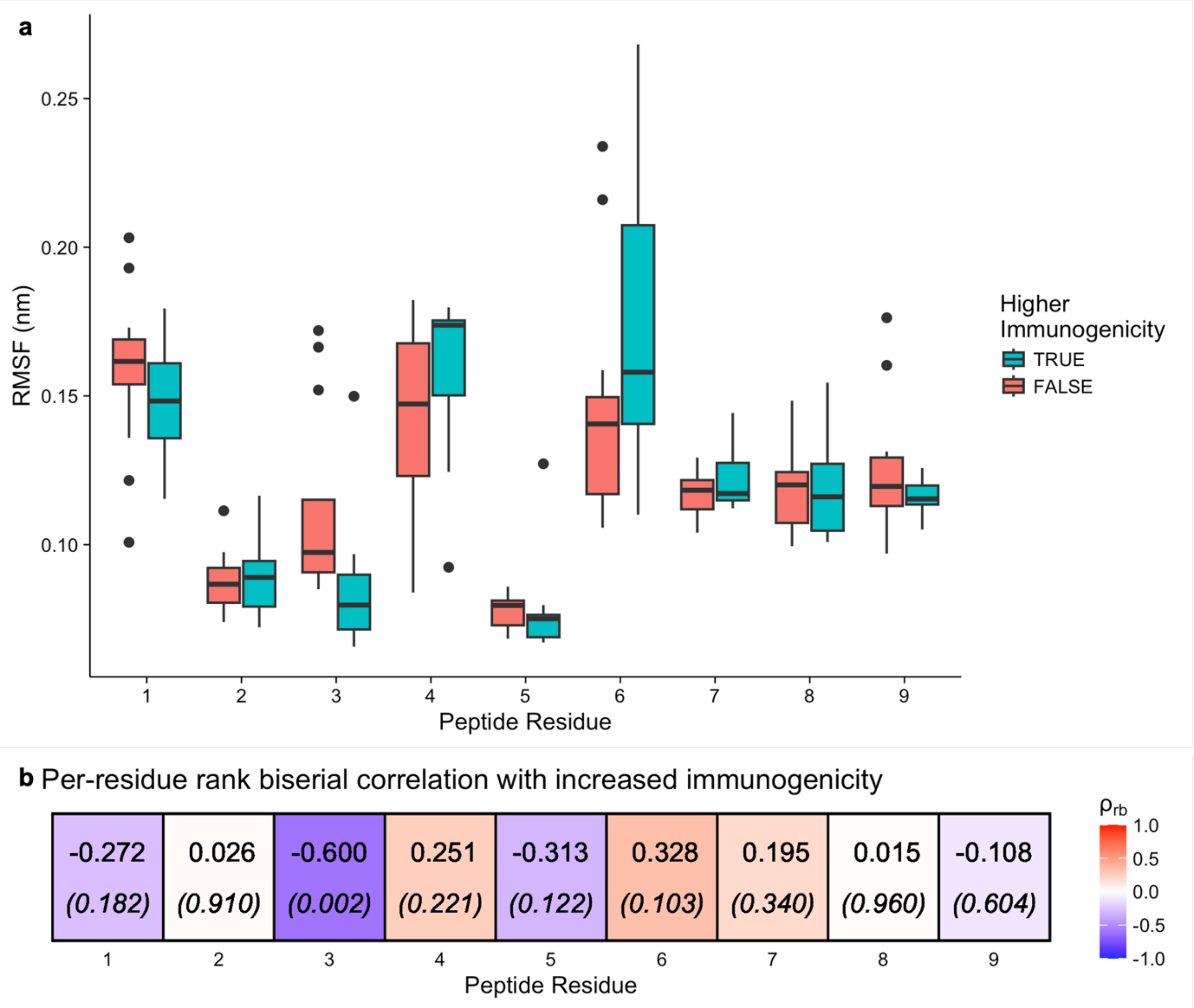

Supplementary Fig. 8. MD-derived per-residue peptide dynamics correlate with immunogenicity. MD trajectories were grouped according to their relative immunogenicity, that is the p3P trajectories were considered as more immunogenic than their paired p3V trajectory counterparts, reflecting previously published findings [1]. **a** Boxplots summarizing the observed RMSF values per trajectory. Visually, p3V/P and p6F display the most striking differences. **b** Rank biserial correlation of per-residue RSMF values and immunogenicity. The uncorrected p-values, obtained via permutation testing, are indicated in parentheses. In this comparison, p3V/P and p6F are also the strongest correlates of immunogenicity.

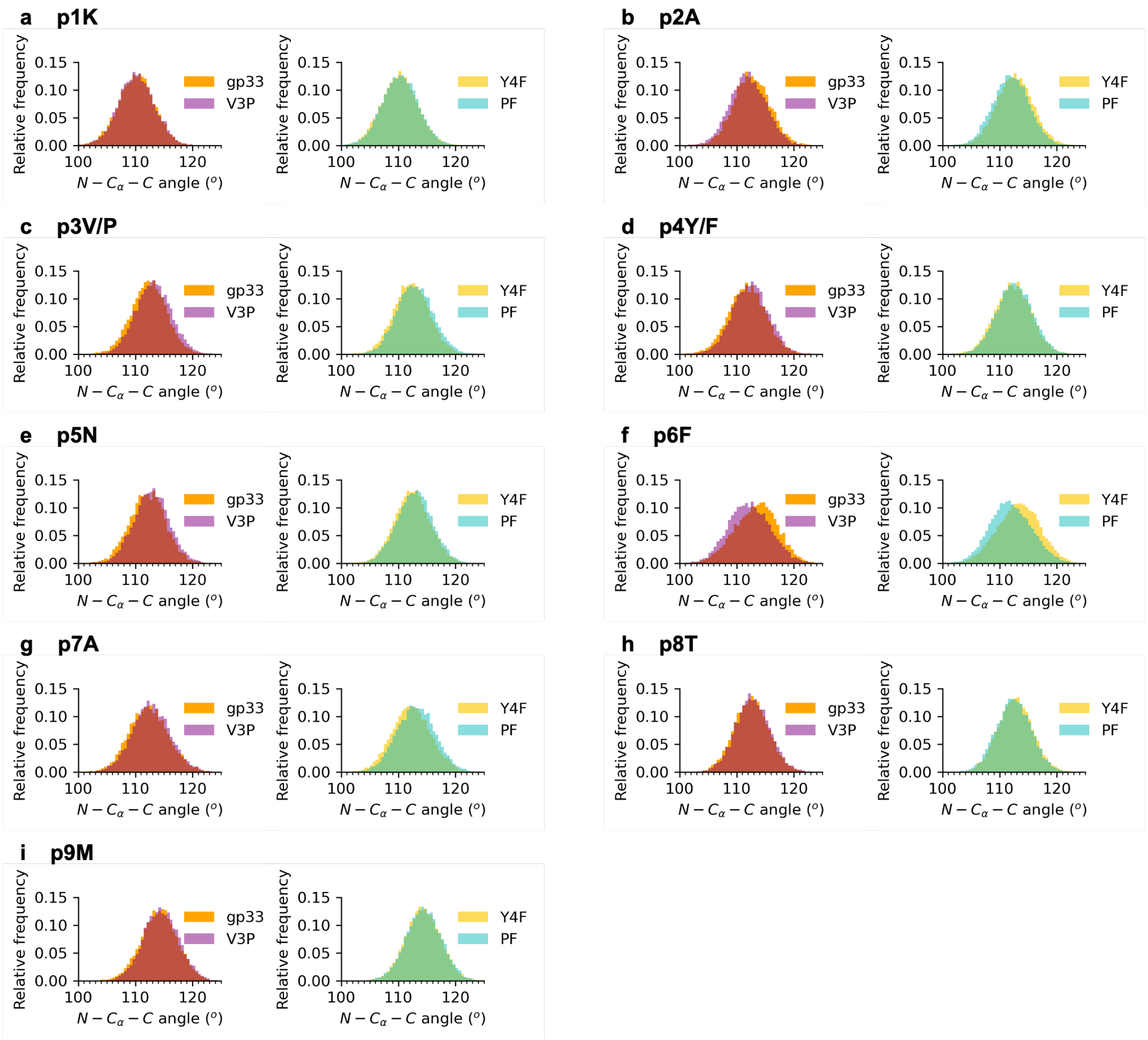

Supplementary Fig. 9. The observed backbone angles (N-C$_\alpha$-C) of each peptide residue throughout the MD trajectories imply that the altered dynamics at p3 are not transmitted to p6 via the peptide backbone. a, **b, c, d, e, f, g, h, i** Relative frequency histograms of the backbone angles of each indicated peptide residue. MD trajectories were grouped according to peptide. Only p6F (**f**) shows a distinct difference in backbone angle when comparing p3V with p3P peptides.

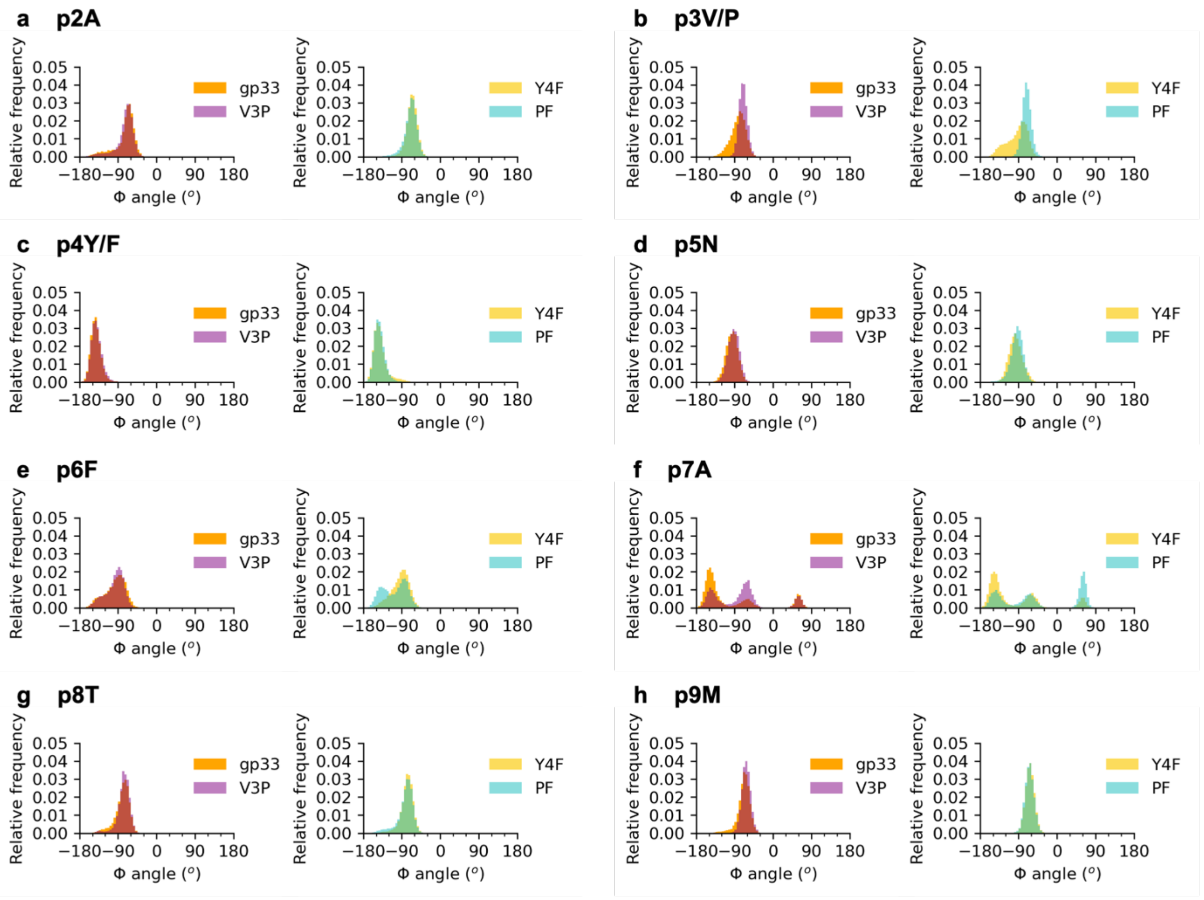

Supplementary Fig. 10. The observed Φ dihedral angles (C-N-C$_\alpha$-C) of each peptide residue throughout the MD trajectories imply that the altered dynamics at p3 are not transmitted to p6 via the peptide backbone. a, **b, c, d, e, f, g, h** Relative frequency histograms of the Φ angles of each indicated peptide residue. MD trajectories were grouped according to peptide. The Φ angles of p3V/P (**b**) and p7A (**f**) show distinct differences in conformational sampling. Most notably, p4Y/F (**c**) and p5N (**d**) remain similar, regardless of the presence of a proline or valine at the third peptide position.

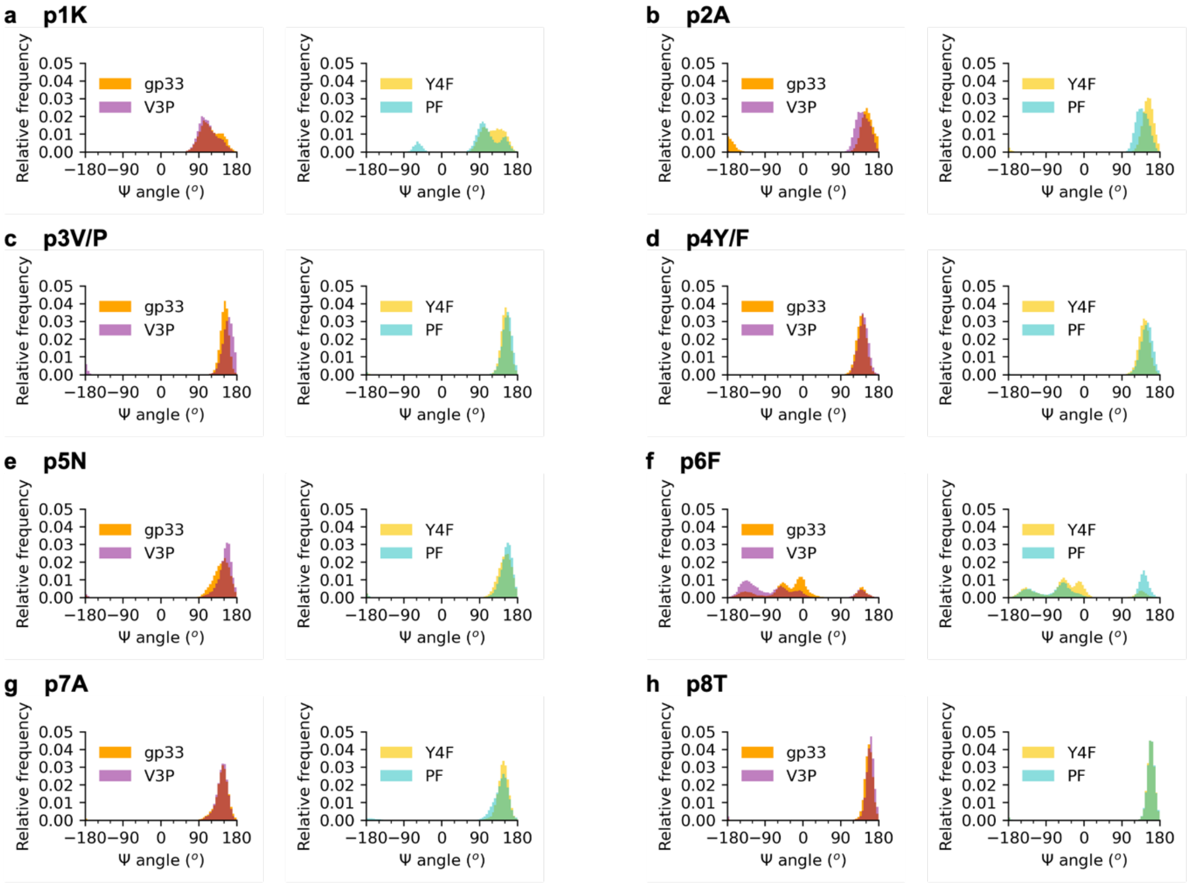

Supplementary Fig. 11 The observed Ψ dihedral angles (N-C$_\alpha$-C-N) of each peptide residue throughout the MD trajectories imply that the altered dynamics at p3 are not transmitted to p6 via the peptide backbone. a, **b, c, d, e, f, g, h** Relative frequency histograms of the Ψ angles of each indicated peptide residue. MD trajectories were grouped according to peptide. The Ψ angles of p2A (**b**) and p6F (**f**) show distinct differences in conformational sampling. Most notably, p4Y/F (**d**) and p5N (**e**) remain similar, regardless of the presence of a proline or valine at the third peptide position.

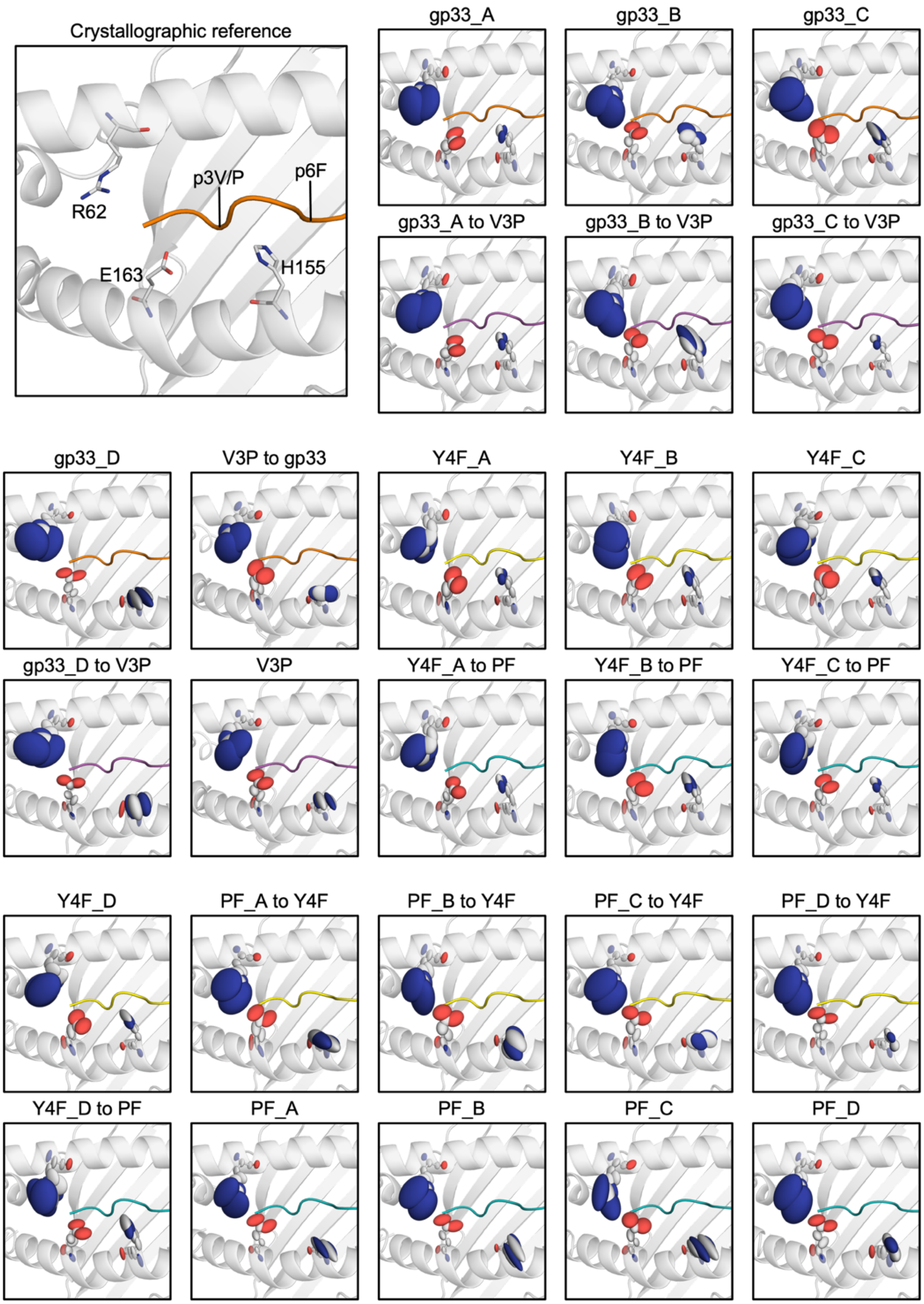

Supplementary Fig. 12. Visual overview of R62, H155, and E163 sidechain dynamics throughout all trajectories. Stick and ellipsoid representation of each sidechain of interest.

Ellipsoids are scaled and shaped according to the anisotropic atomic temperature factors derived from each trajectory and mapped to their respective crystallographic models. The backbone of H-2D^b (white), gp33 (orange), V3P (purple), Y4F (yellow), and PF (teal) are shown in cartoon representation. Trajectories are paired vertically, one derived from the initial crystallographic model and the other derived from an *in silico* mutation between p3V and p3P. No relationship between the p3P substitution and R62 sidechain dynamics is visually evident. On average, increased conformational sampling of H155 is observed in the presence of p3P. In the trajectories where H155 is more dynamic, p6F is generally more dynamic (**Fig. 2, Supplementary Fig. 7**). E163, which is near p3 in the structure, displays smaller fluctuations in the presence of p3P compared to p3V.

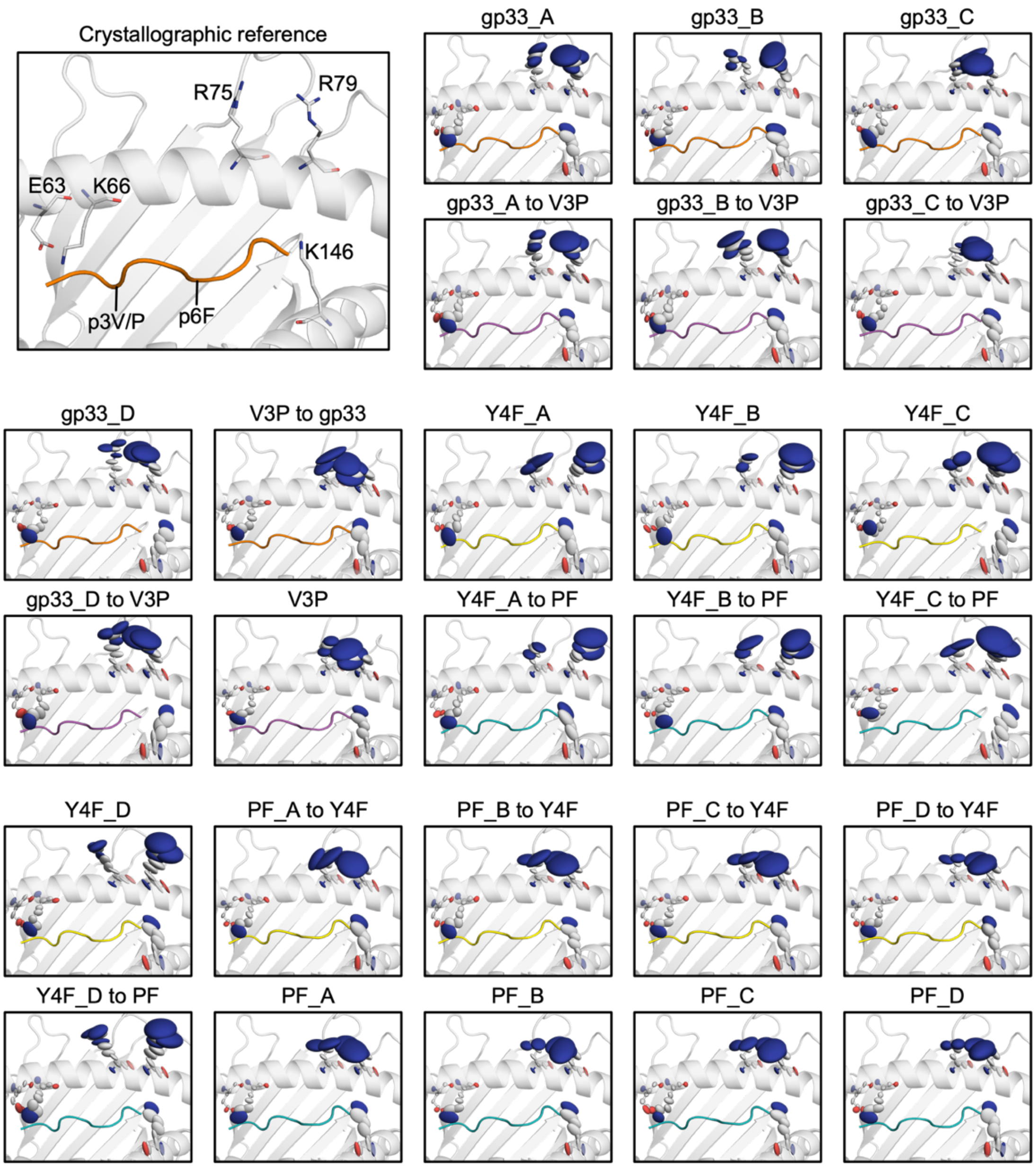

Supplementary Fig. 13. Visual overview of E63, K66, R75, R79 and K146 sidechain dynamics throughout all trajectories. Stick and ellipsoid representation of each sidechain of interest. Ellipsoids are scaled and shaped according to the anisotropic atomic temperature factors derived from each trajectory and mapped to their respective crystallographic models. The backbone of H-2D^b (white), gp33 (orange), V3P (purple), Y4F (yellow), and PF (teal) are shown in cartoon representation. Trajectories are paired vertically, one derived from the initial crystallographic

model and the other derived from an *in silico* mutation between p3V and p3P. Visually, no consistent differences in sidechain fluctuations for these residues of interest when comparing p3V and p3P trajectories were identified.

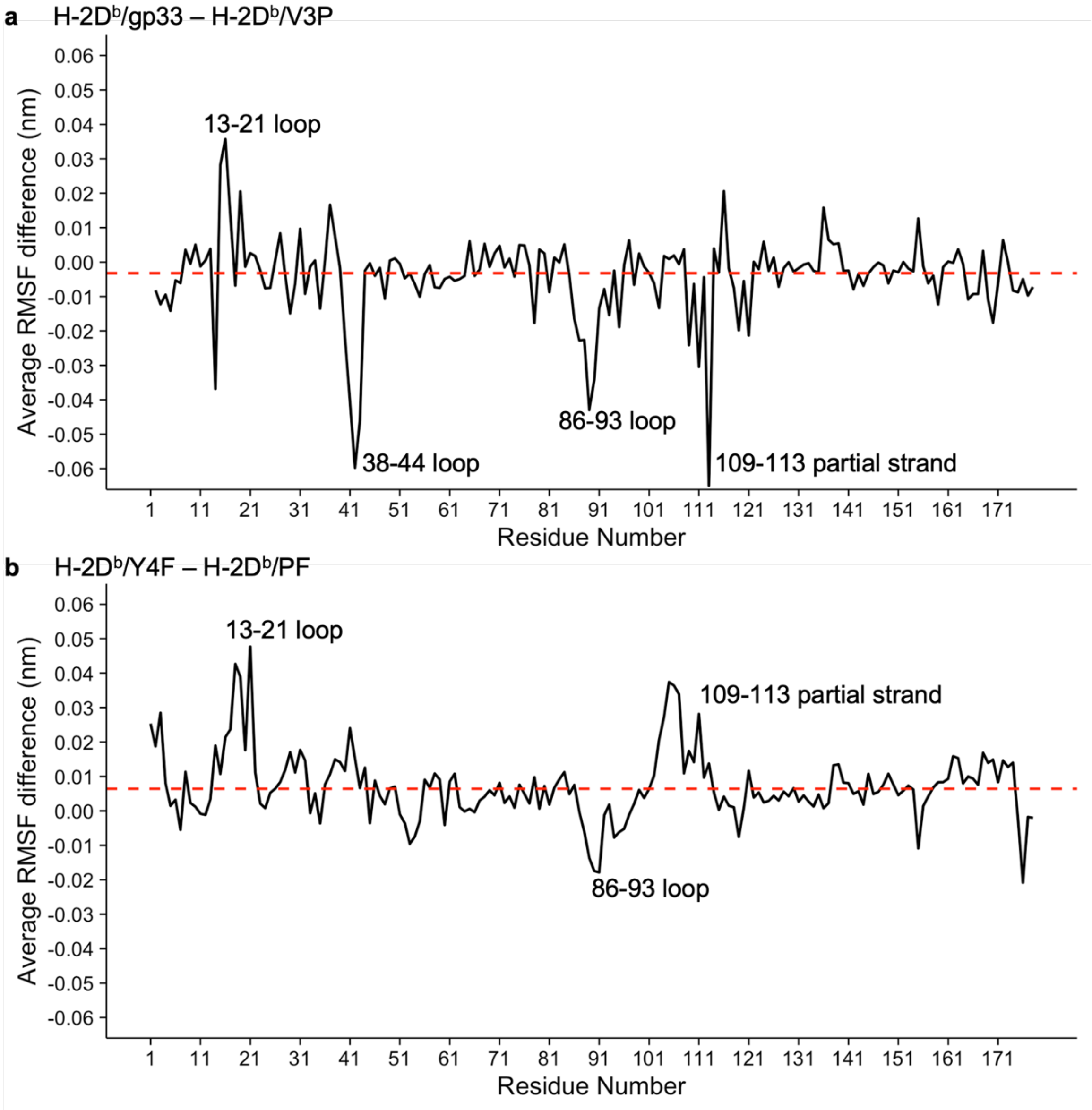

Supplementary Fig. 14. Differences in average per-residue RMSF values for H-2D^b. MD trajectories were grouped according to peptide to obtain the average per-residue RMSF. The difference in average per-residue RMSF was calculated by subtracting the values of comparable trajectories: **a** H-2D^b/gp33 – H-2D^b/V3P and **b** H-2D^b/Y4F – H-2D^b/PF. Dashed red lines denote the average difference. The regions of interest identified in ensemble refinements (**Supplementary Figs. 2, 3**) are labeled.

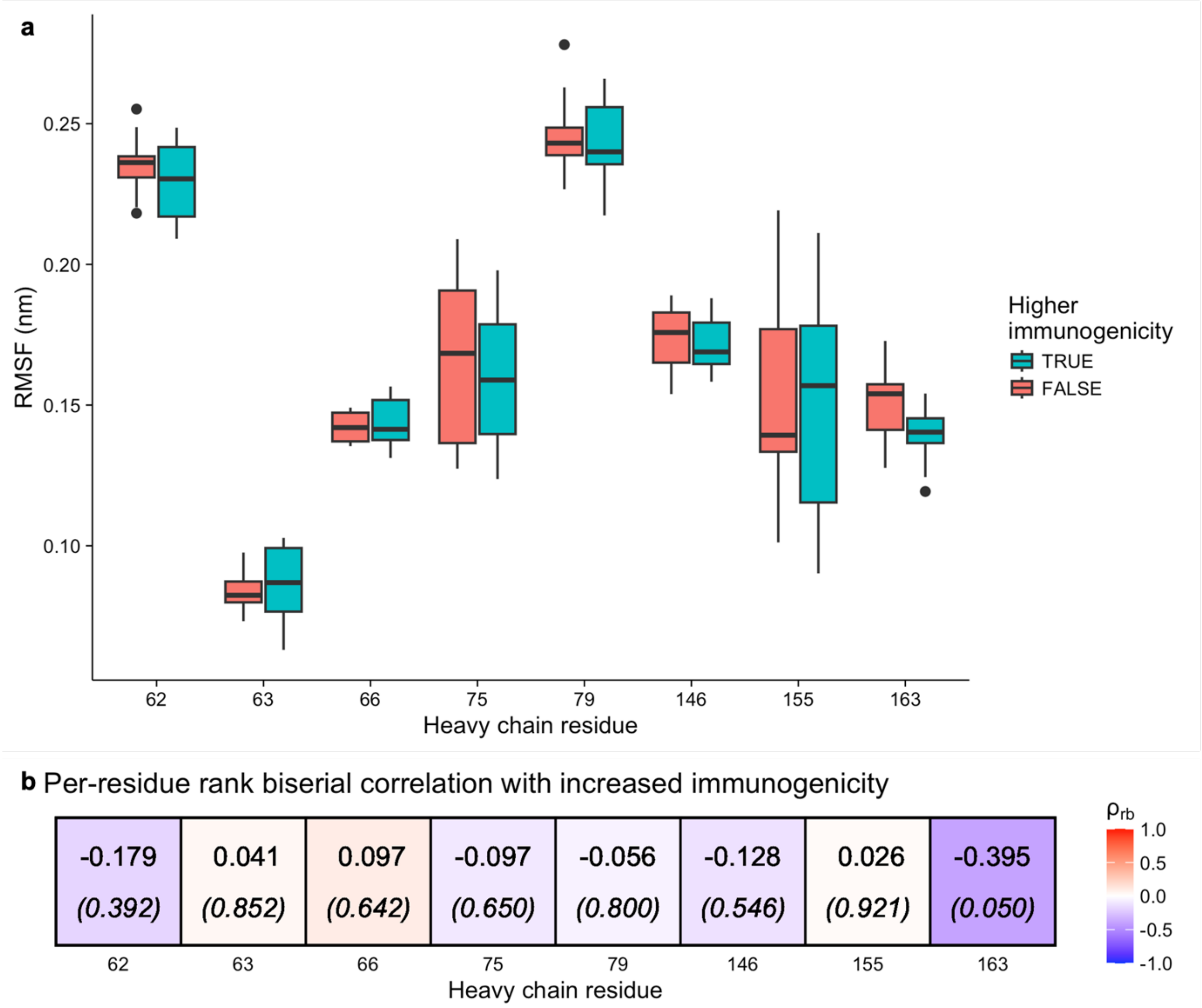

Supplementary Fig. 15. Assessing the correlation of H-2D^{b} dynamics with immunogenicity. MD trajectories were grouped according to their relative immunogenicity, that is the p3P trajectories were considered more immunogenic than their paired p3V trajectory counterparts, reflecting previously published findings [1]. **a** Boxplots summarizing the observed RMSF values per trajectory. Visually, E163 displays the largest difference. **b** Rank biserial correlation of per-residue RSMF values and immunogenicity. The uncorrected p-values, obtained via permutation testing, are indicated in parentheses. In this comparison, E163 is also the strongest correlate of immunogenicity.

Supplementary Table 4. Residues that are significantly correlated with each peptide position.

Table summarizes identified significant correlates in **Fig. 3**.

Peptide residue	Significantly correlated residues
p1K	R121 E163
p2A	S4 M5 Y7 A11 Y45 E58 R62 E63 K66 G69 S99 G100 C101 D102 G112 G120 K157 A158 Y159 L160 E161 C164 p3V/P
p3V/P	G16 L160 p2A
p4Y/F	–
p5N	W73 L114 F116 A117 I124 L130 K131 T132 W133 T134 R145 W147 E148 S150 G151 A152 A153 E154 Y156 p7A p8T p9M
p6F	H155 R170 K173 T178
p7A	W73 L114 F116 A117 E119 D122 Y123 I124 A125 L126 E128 D129 L130 K131 T132 W133 T134 A135 A140 A141 I142 T143 R144 R145 K146 W147 E148 Q149 S150 G151 A152 A153 E154 Y156 K157 p5N p8T p9M
p8T	W73 V76 S77 F116 A117 Y118 D122 Y123 I124 A125 E128 D129 T132 W133 T134 A135 A140 Q141 I142 T143 R144 R145 K146 W147 E148 Q149 S150 A152 p5N p7A p9M
p9M	P47 A49 W73 V76 S77 N80 L81 Y84 L103 F116 A117 Y118 D122 Y123 I124 A125 W133 T134 A135 D137 M138 A139 A140 Q141 I142 T143 R144 R145 K146 W147 E148 S150 G151 A152 p5N p7A p8T

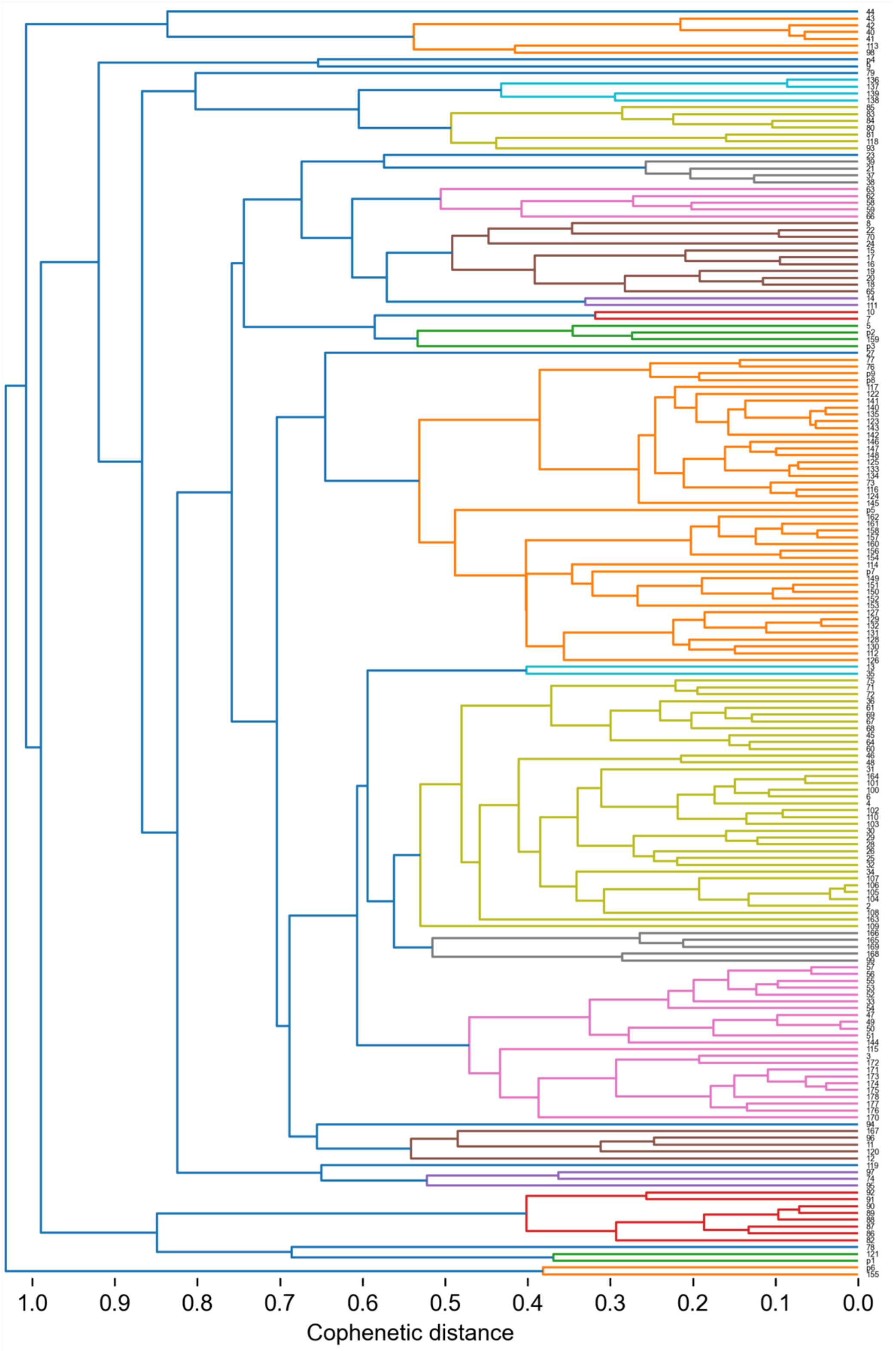

Supplementary Fig. 16. Agglomerative hierarchical clustering dendrogram derived from the raw Spearman rank correlations of per-residue RMSF values. The dendrogram was

constructed using the UPGMA algorithm, using pairwise correlation distance as the distance metric. Dendrogram leaves are clustered and colored using a cophenetic distance threshold of 0.55, a distance which allows p3V/P to be a member of one of the clusters.

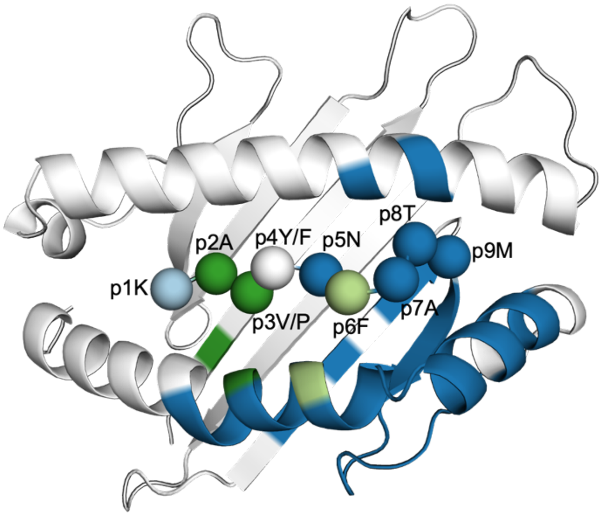

Supplementary Fig. 17. Clusters from agglomerative hierarchical clustering containing peptide residues mapped back onto the structure of H-2D^{b}/gp33. In this mapping, p3V/P and p2A can be seen to cluster with Y159 on the α2 helix and M5 on the β1 strand. Both p5N and p7A form part of a larger cluster including the neighboring regions around Y159 on the α2 helix. H155 and p6F form their own cluster.

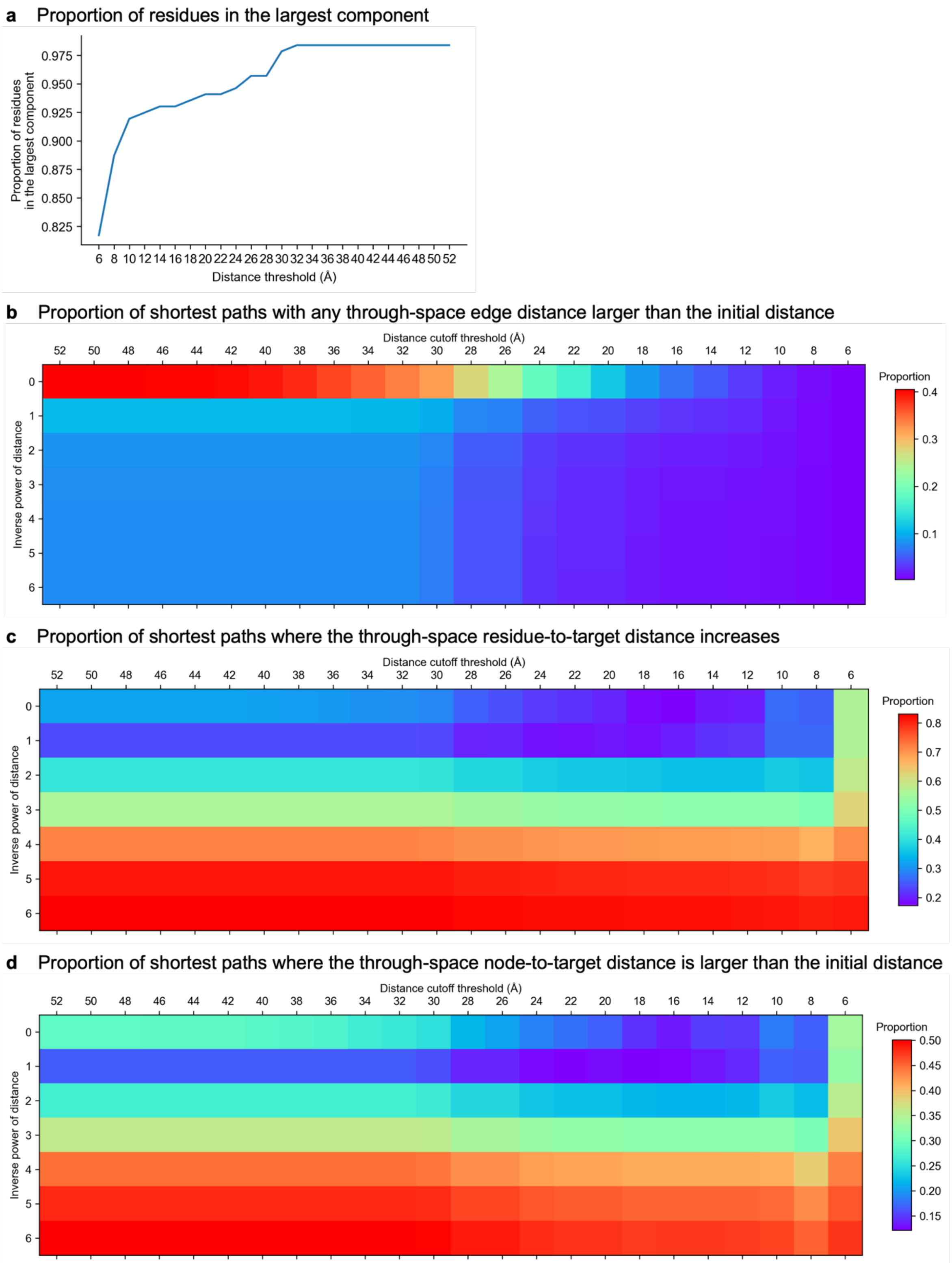

Supplementary Fig. 18. Overview of the network validation statistics used to determine which edge weight metric yielded the most biophysically relevant network. A 2D scan of

different distance cutoff thresholds and inverse powers of distance for network construction was performed. Note that a distance threshold of 52 Å is larger than any pairwise through-space C_α distance at the interface (51.1 Å). **a** Proportion of residues that remain in the largest connected component of the network using different distance thresholds. **b** Proportion of shortest paths where any through-space edge distance along the path is greater than the initial distance between the start and end nodes (F_1). The biophysically relevant network was chosen by minimizing edge weight complexity and **b** while maximizing **a** (>90%). **c** Proportion of shortest paths where the through-space node-to-target distance increases (F_2). **d** Proportion of shortest paths where any through-space node-to-target distance along the path is larger than the initial distance (F_3).

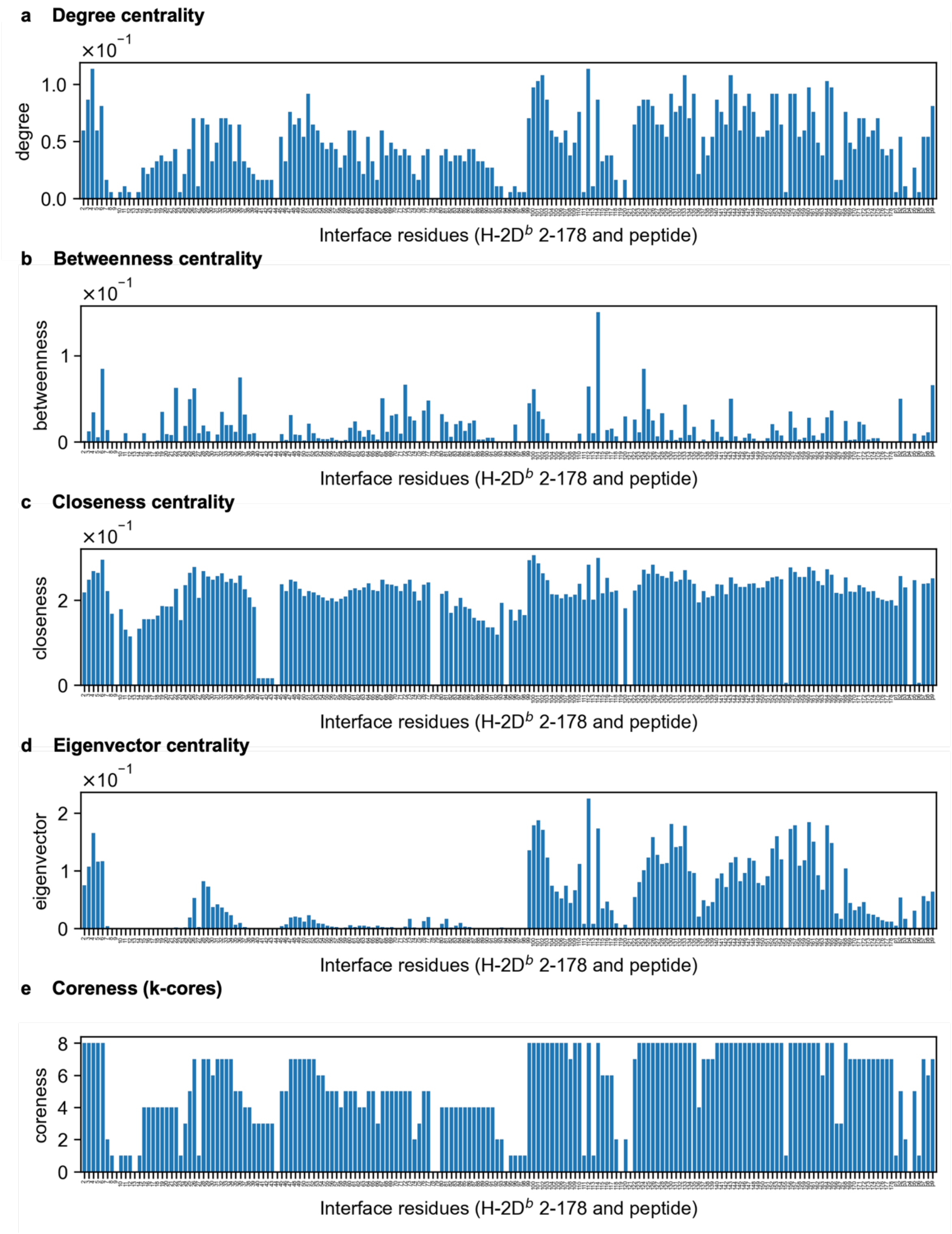

Supplementary Fig. 19. Centrality measures for each residue in the biophysically relevant network. a, **b**, **c**, **d**, **e** The different centrality measures presented in **Fig. 4** are plotted accordingly for reference.

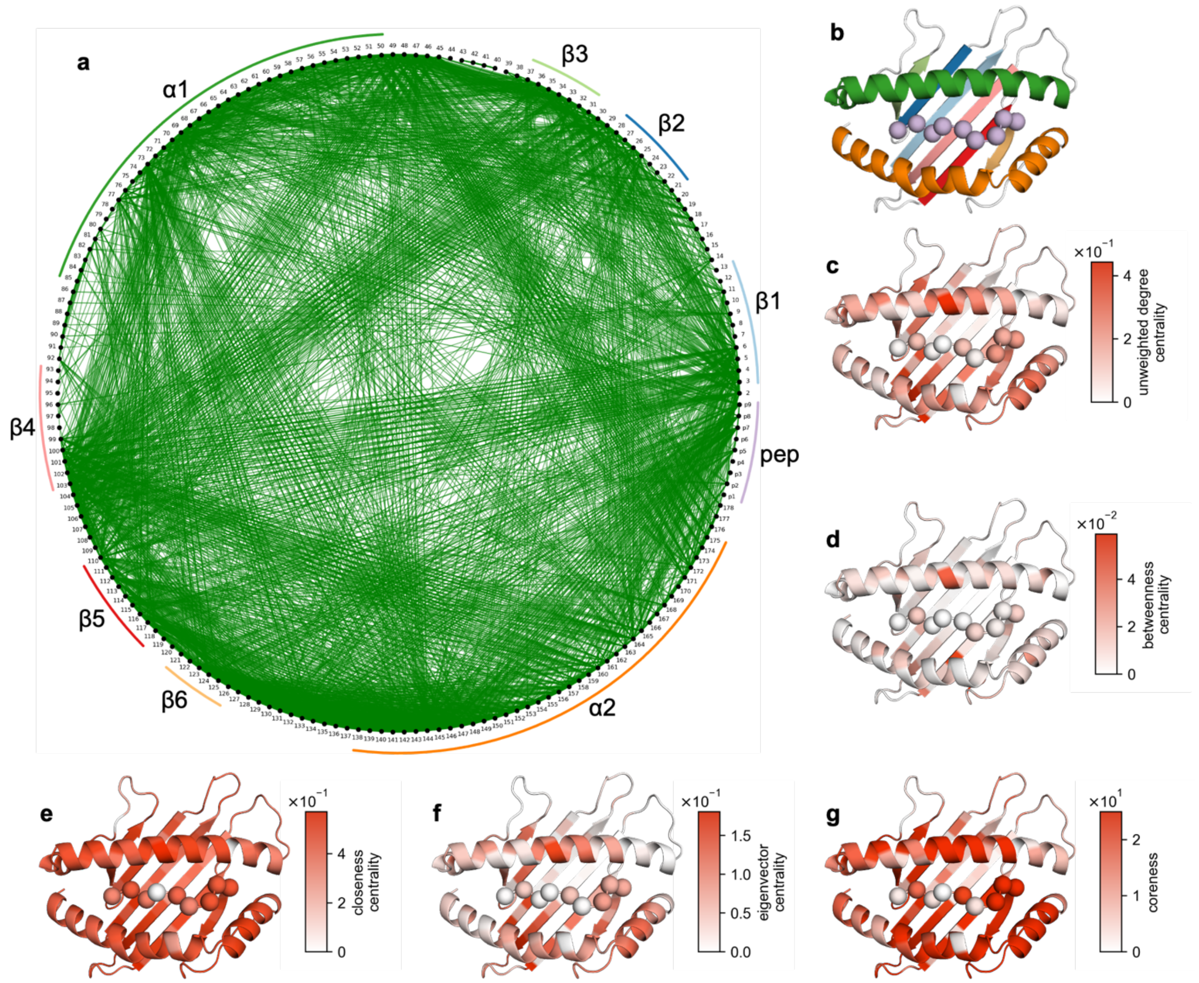

Supplementary Fig. 20. Network generated from the correlation matrix without implementing inter-residue through-space distance thresholds. a The network encoding interface dynamics, where each node (black) represents an interface residue and edges (green) are significant correlations between residues whose alpha-carbon distance does not surpass 10 Å. Edge transparency and width are scaled according to $|\rho_S|$. Secondary structure features of the interface have been labeled. **b** Secondary structure features of the H-2D^b/peptide interface are colored according to the same label colors as in **a**. Various informative network centrality measures can be mapped to structure: **c** unweighted degree centrality, **d** betweenness centrality, **e** closeness centrality, **f** eigenvector centrality, and **g** coreness. Plots showing centrality measures per residue are provided (**Supplementary Fig. 21**).

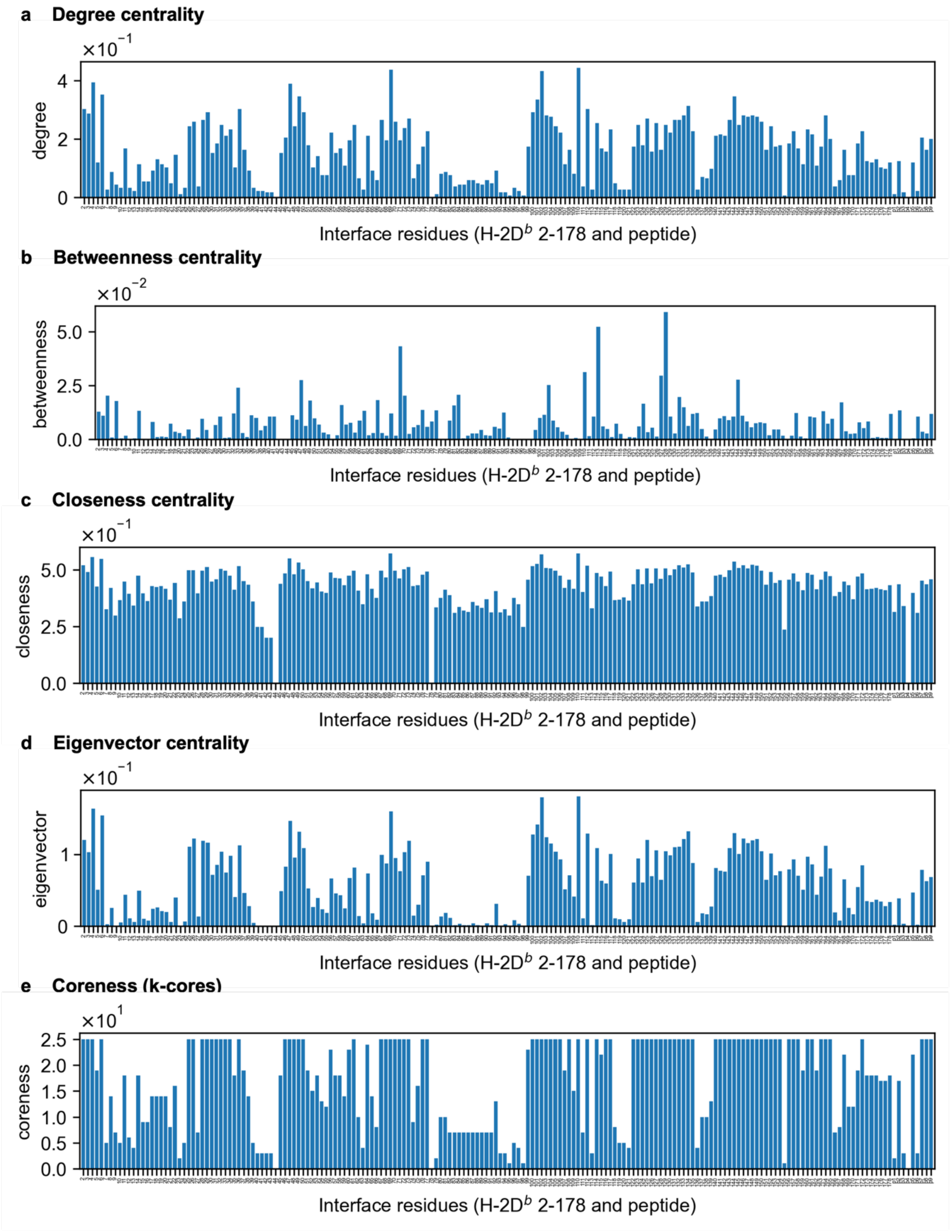

Supplementary Fig. 21. Centrality measures for each residue in the correlation network. a, b, c, d, e The different centrality measures presented (**Supplementary Fig. 20**) plotted accordingly for reference.

References

1. Duru, A. D. *et al.* Tuning antiviral CD8 T-cell response via proline-altered peptide ligand vaccination. *PLoS Pathog* **16**, e1008244 (2020).
